# Supplementary material for: Photooxidation of 2-(tert-Butyl)-3-Methyl-2,3,5,6,7,8-Hexahydroquinazolin-4(1H)-one, an Example of Singlet Oxygen ene Reaction
Source: Molecules. 2020 Oct 29;25(21):5008. doi: 10.3390/molecules25215008 (PMC7662339; doi:10.3390/molecules25215008)
Supplement: Supplementary file 1 [file molecules-25-05008-s001.pdf]

Photooxidation of 2-(*tert*-butyl)-3-methyl-2,3,5,6,7,8-hexahydroquinazolin-4(*1H*)-one, an example of singlet oxygen *ene* reaction.

A. Méndez[1], Jonathan R. Valdez-Camacho[1], and Jaime Escalante[1,*]

[1] The Center for Chemical Research, Autonomous University of Morelos State, Avenida Universidad 1001, Chamilpa, Cuernavaca 62210, Mexico.

* Correspondence: jaime@uaem.mx (J.E.);
Tel.: +52-77-7329-7997 (J.E.)

Supporting Information

Table of contents:

		Page
1.	General considerations for the synthesis and characterizations	2
2.	Synthesis of 2-(*tert*-butyl)-3-methyl-2,3-dihydroquinazolin-4(*1H*)-one (8)	3
3.	Synthesis of 2-(*tert*-butyl)-3-methyl-2,3,5,6,7,8-hexahydroquinazolin-4(*1H*)-one (11)	3
4.	Synthesis of 2-(*tert*-butyl)-4a-hydroperoxy-3-methyl-2,4a,5,6,7,8-hexahydro-quinazolin-4(*3H*)-one (12)	4
5.	Synthesis of 1-acetyl-2-(*tert*-butyl)-3-methyl-2,3,5,6,7,8-hexahydroquinazolin-4(*1H*)-one (13)	4
6.	^{1}H and ^{13}C NMR spectra for compounds 8	5
7.	^{1}H and ^{13}C NMR spectra for compounds 11	6
8.	^{1}H and ^{13}C NMR spectra for compounds 12	7
9.	^{1}H and ^{13}C NMR spectra for compounds 13	8
10.	Crystallographic data and structure refinement for product 8	9
11.	Crystallographic data and structure refinement for product 12	13
12.	Crystallographic data and structure refinement for product 13	21
13.	Cartesian coordinates from optimized structures of 11	26
14.	Cartesian coordinates from optimized structures of 12	28
15.	Cartesian coordinates from optimized structures of R	30
16.	Cartesian coordinates from optimized structures of P	32
17.	Cartesian coordinates from optimized structures of $TS1_Z$	34
18.	Cartesian coordinates from optimized structures of $TS1_D$	36
19.	Cartesian coordinates from optimized structures of $TS2_Z$	38
20.	Cartesian coordinates from optimized structures of $TS2_D$	40
21.	Cartesian coordinates from optimized structures of I_Z	42
22.	Cartesian coordinates from optimized structures of I_D	44
23.	Supplementary References	45

1) General considerations for the synthesis and characterizations

Dichloromethane, ethyl acetate, and hexane were distilled before use. Toluene (Aldrich), acetonitrile (Merck), tert-butanol (Aldrich), Isatoic Anhydride (Aldrich), Methylamine (Aldrich), p-toluene sulfonic acid (Aldrich), pivalaldehyde (Aldrich), sodium bicarbonate (Aldrich), sodium sulfate (Aldrich), sodium (Aldrich), palladium/carbon (Aldrich), 4,4-dimethylamino pyridine (Aldrich), and acetyl chloride (Aldrich) were acquired from the commercial suppliers indicated in parenthesis and used without further purification. Reactions were monitored by TLC on Al plates coated with silica gel with fluorescent indicator (60 F_{254}). Column chromatography (CC) was performed on silica gel (230-400 mesh Merck). NMR Spectra: Varian Gemini at 200 (^{1}H) and 50 MHz (^{13}C), Varian Inova at 400 (^{1}H) and 100 MHz (^{13}C), Bruker AVANCE III HD 500 MHz (^{1}H) and 125 MHz (^{13}C). Chemical shifts in the ^{1}H NMR spectra are reported in parts per million (ppm, δ scale) downfield relative to tetramethylsilane (δ 0), which was used as an internal standard in $CDCl_3$, or relative to the residual proton in the solvent ($CDCl_3$: δ 7.26). Chemical shifts in the ^{13}C NMR spectra are reported in parts per million (ppm, δ scale) downfield relative to tetramethylsilane, and are referenced to the ^{13}C NMR resonance of the solvent ($CDCl_3$: δ 77.23). A mass spectrometric analysis was performed using an Agilent 6530 Quadrupole Time of Flight (QTOF) LCMS with an electrospray ionization (ESI) source (Agilent Technologies, Santa Clara, CA, USA). A mass spectrometry analysis was conducted in positive ion mode, set for a detection of mass-to-charge ratio (m/z) of 100 to 1000. Intensity data for the X-ray crystallographic analyses were collected at 100 or 120 K on a Agilent Technologies Super Nova equipped with a CCD area detector (EosS2). Using Mo-*K*a (λ = 0.7107 Å). The structures were solved with the OLEX2 [1] program package using SHEXLTL [2] and refined with SHELXL-2014 [3]. Hydrogen atoms were generated in calculated positions and constrained with the use of a riding model. The crystal structure data were deposited at the Cambridge Crystallographic Data Center.

2) Synthesis of 2-(*tert*-butyl)-3-methyl-2,3-dihydroquinazolin-4(*1H*)-one (8) [4]

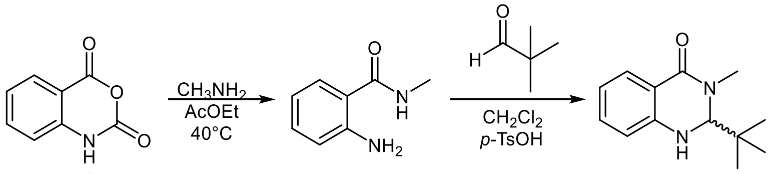

*Scheme 1. Synthesis of quinazolinone **8***

Prepared according to a known procedure [4] from isatoic anhydride (6 g, 30.68 mmol), methylamine (18.24 mL, 122.72 mmol), and ethyl acetate (50 mL). The reaction was heated at 40 ℃ for 2h. The resulting material was filtered and concentrated in a rotavapor, producing 2.06g of reaction crude. A suspension of reaction crude (2.06 g), *p*-toluenesulfonic acid (0.103 g, 5% w/w), pivalaldehyde (1.86 mL, 1.2 equiv.), and dichloromethane (60 mL). The reaction was refluxed for 5 h. The resulting material was filtered and concentrated in a rotavapor, then purified by column chromatography on silica, eluting with hexane-ethyl acetate (9:1 to 6:4). Yield: 90%; white solid; mp: 145-146 ℃; ^{1}H NMR ($CDCl_3$, 200 MHz): δ 0.91 (s, 9H), 3.20 (s, 3H), 4.34(d, 1H), 4.71 (sa, 1H), 6.56-6.58 (d, 1H), 6.70-6.74 (t, 1H), 7.19-7.23 (t, 1H), 7.80-7.83 (d, 1H). ^{13}C NMR ($CDCl_3$, 100 MHz): δ 26.3 (C10-12), 38.5 (C11), 41.9 (C9), 79.9 (C2), 113.1 (C8), 116.6 (C4a), 118.1 (C6), 128.1 (C5), 133.4 (C7), 146.5 (C8a), 163.8 (C4). HREIMS m/z 218.1440 (calculated for $C_{13}H_{18}N_20$, 218.1419). CCDC No. 2006915.

3) Synthesis of 2-(*tert*-butyl)-3-methyl-2,3,5,6,7,8-hexahydroquinazolin-4(*1H*)-one (11).

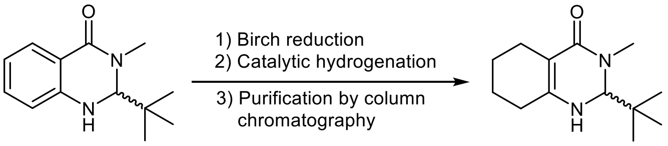

*Scheme 2. Synthesis of hexahydroquinazolinone **11.***

Prepared from ammonia (50 mL), 2-(*tert*-butyl)-3-methyl-2,3-dihydroquinazolin-4(1H)-one **8** (1 g, 4.64 mmol), and sodium (0.644 g, 28 mmol). The reaction was cooled and stirred at -78 ℃. After 30 minutes, *tert*-butanol (1.4 mL, 14 mmol) was added and was allowed react for 1 hour. Finally, the reaction is quenched adding ammonium chloride (0.64 g, 18.56 mmol). The ammonia is evaporated and reaction crude is dissolved in dichloromethane, dried with sodium sulfate and the excess solvent is concentrated in vacuo. A suspension of reaction crude (0.8 g), Pd/C (0.04g, 5% w/w), and methanol (60 mL) was placed in a 100 mL flask containing a stir bar, then the system was closed with a septum. Two balloons were placed with hydrogen and the reaction was stirred overnight. The reaction mixture was filtered and concentrated in a rotavapor rotavapor, then purified by column chromatography on silica, eluting with hexane-ethyl acetate (9:1 to 2:8). Yield: 28%; light-yellow solid; mp: 134-137 ℃. ^{1}H NMR (200 MHz, $CDCl_3$) δ 4.18 (d, 1H, J = 4.6 Hz, 1H), 3.88 (s, 1H), 3.06 (s, 3H),

2.27 – 2.04 (m, 4H), 1.77 – 1.36 (m, 4H), 0.91 (s, 9H). ^{13}C NMR due to the high reactivity of **11**, it was not possible to acquire a suitable spectrum for its assignment.

4) Synthesis of 2-(*tert*-butyl)-4a-hydroperoxy-3-methyl-2,4a,5,6,7,8-hexahydroquinazolin-4(*3H*)-one (12)

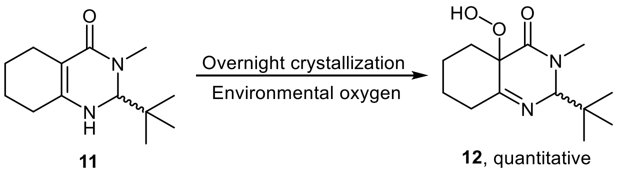

*Scheme 3. Crystallization of product **12**.*

2-(*tert*-Butyl)-3-methyl-2,3,5,6,7,8-hexahydroquinazolin-4(1H)-one **11** (0.071g, 0.32 mmol) was dissolved in 2 mL dichloromethane inside a suitable test tube, then 8 mL of hexane was added carefully to form a biphase, the system was closed with a cotton septum and left exposed to the environment for the evaporation of the solvents. After overnight, crystals of the expected compound were obtained. Yield: 81mg, quantitative yield; White crystal; mp: 152-154 ℃; ^{1}H NMR (500 MHz, CDCl3) δ 1.02 (s, 9H), 1.55-1.67 (m, 3H), 1.94-1.97 (m, 1H), 2.08-2.11 (m, 1H), 2.39-2.47 (m, 2H), 2.94-2.95 (m, 1H) 3.10 (s, 3H), 4.90 (s, 1H). ^{13}C NMR (125 MHz, CDCl3) δ 170.3, 168.6, 85.3, 76.3, 40.1, 37.0, 34.2, 33.7, 28.5, 27.2, 20.5. HREIMS m/z 254.1654 (calculated for C13H22N203, 254.1630). CCDC No. 2006916.

5) Synthesis of 1-acetyl-2-(*tert*-butyl)-3-methyl-2,3,5,6,7,8-hexahydroquinazolin-4(*1H*)-one (13)

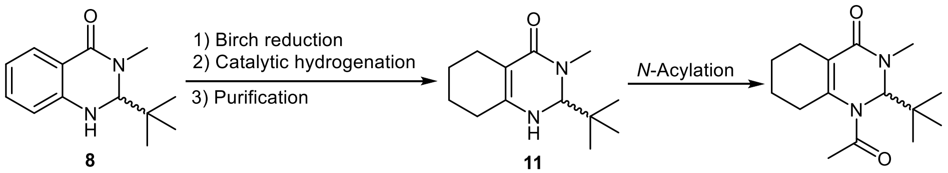

Scheme 4. Protection of N-H group

2-(tert-butyl)-3-methyl-2,3,5,6,7,8-hexahydroquinazolin-4(1H)-one (**11**, 0.8 g, 3.6 mmol), 4-dimethylaminopyridine (0.439 g, 3.6 mmol) and toluene/acetonitrile solution 9:1 (40 mL) was added to a 100 mL flask. It was placed in an ice bath and the system purged with nitrogen. Acetyl chloride (0.3 mL, 4.32 mmol) was added dropwise and the reaction was stirred overnight. After time the reaction crude was filtered and concentrated in a rotavapor then purified by column chromatography on silica, eluting with hexane-ethyl acetate (9:1 to 3:7). Yield: quantitative; white solid; mp**:** 146-148 ℃; ^{1}H NMR (200 MHz, $CDCl_3$) δ 5.44 (s, 1H), 3.03 (s, 3H), 2.79 – 2.54 (m, 2H), 2.13 (s, 3H), 2.09 – 1.98 (m, 2H), 1.91 – 1.36 (m, 4H), 0.89 (s, 9H). ^{13}C NMR (50 MHz $CDCl_3$) δ 170.8, 163.5, 122.3, 38.4, 37.1, 30.5, 27.17, 27.0, 24.1, 22.6, 22.5, 21.6. HREIMS m/z 264.1843 (calculated for $C_{15}H_{24}N_{2}0_{2}$, 264.1838). CCDC No. 2007030.

6) ^{1}H and ^{13}C NMR spectra for compounds 8.

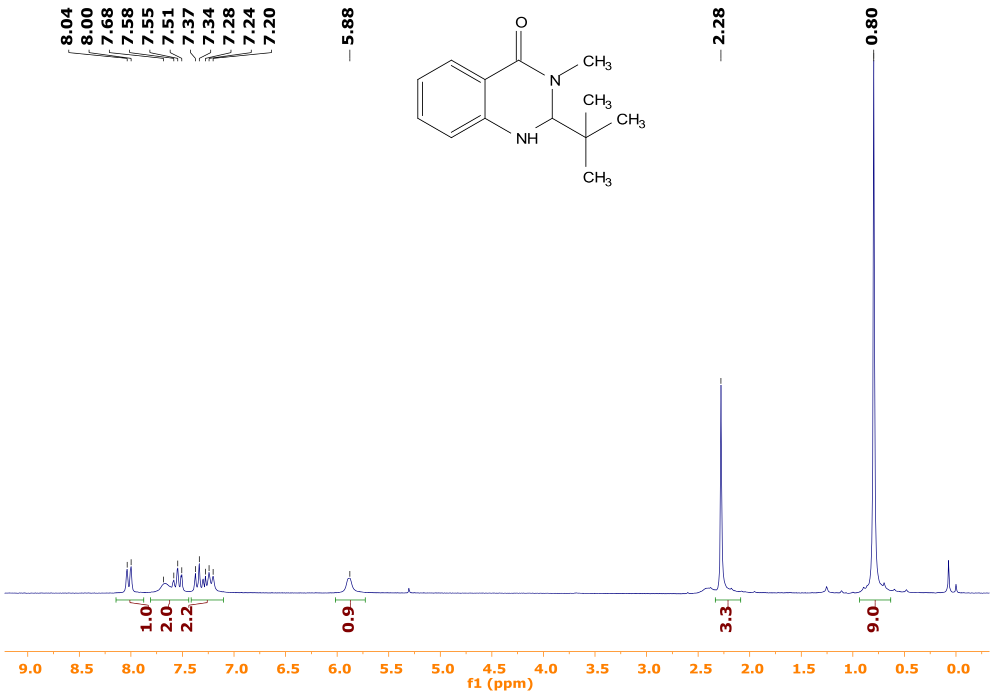

*Figure S1. ^{1}H spectrum (200 MHz, $CDCl_3$) of **8***

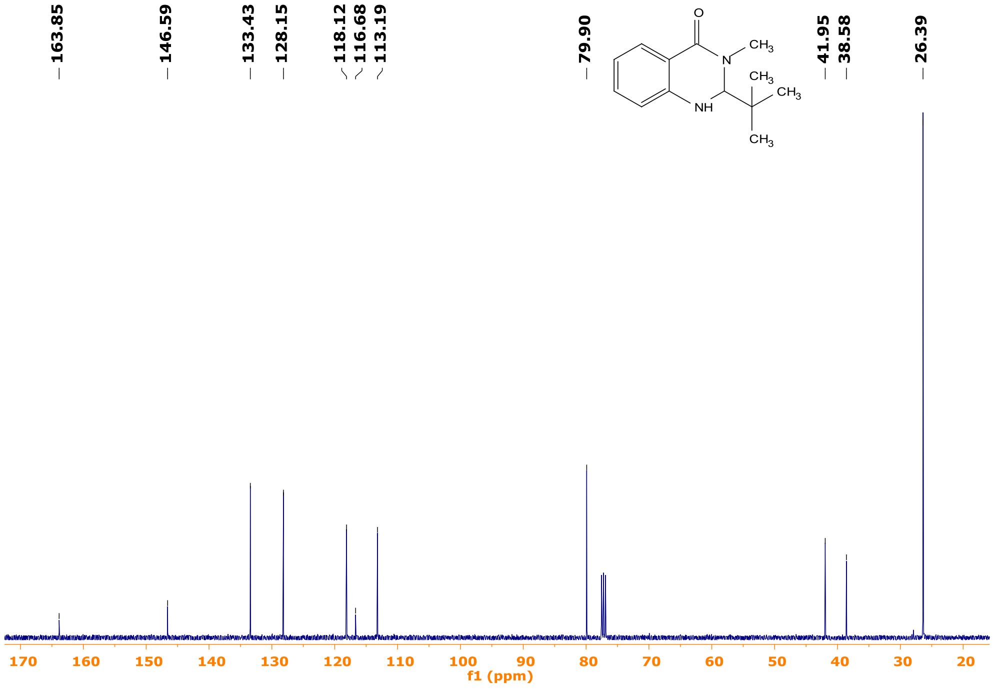

*Figure S2. ^{13}C spectrum (100 MHz, $CDCl_3$) of **8***

7) ^{1}H and ^{13}C NMR spectra for compounds 11.

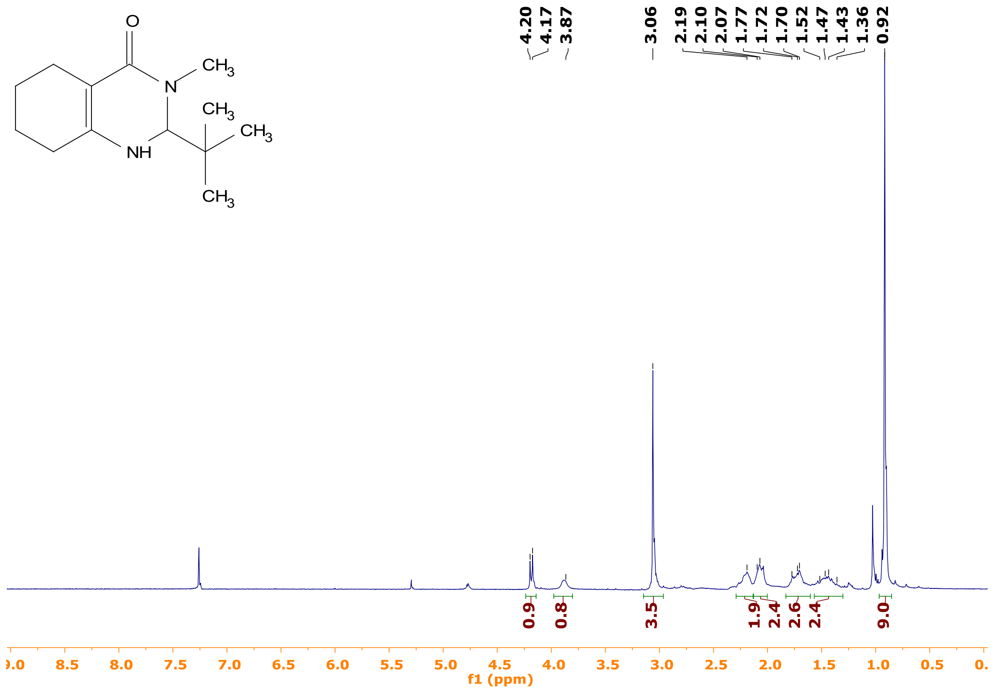

Figure S3. ^{1}H spectrum (200 MHz, $CDCl_3$) of **11**

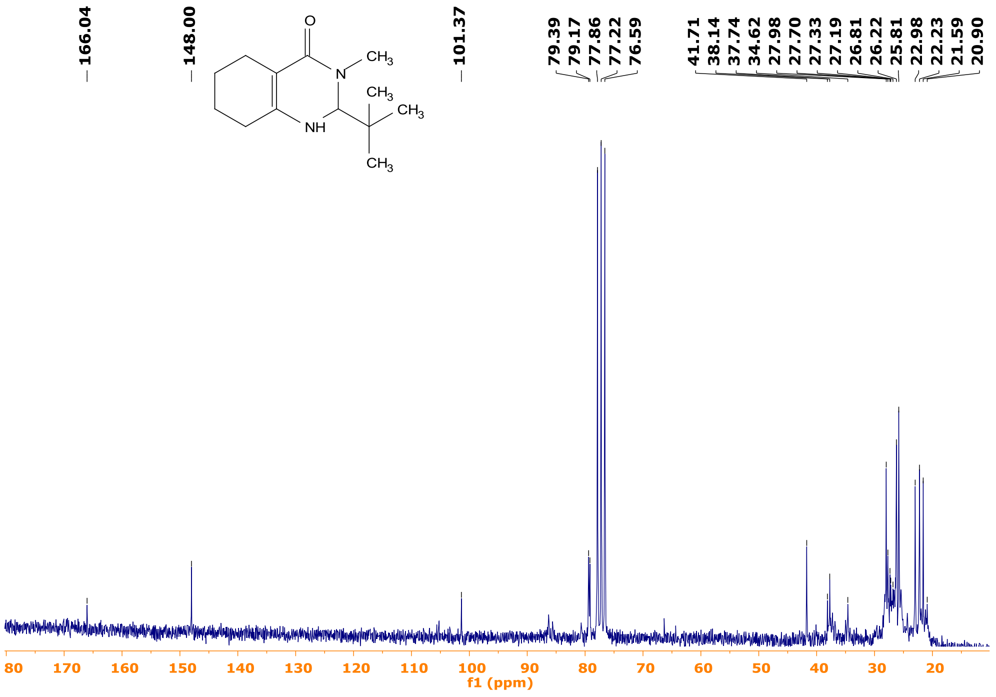

Figure S4. ^{13}C spectrum (50 MHz, $CDCl_3$) of **11**

8) ^{1}H and ^{13}C NMR spectra for compounds 12.

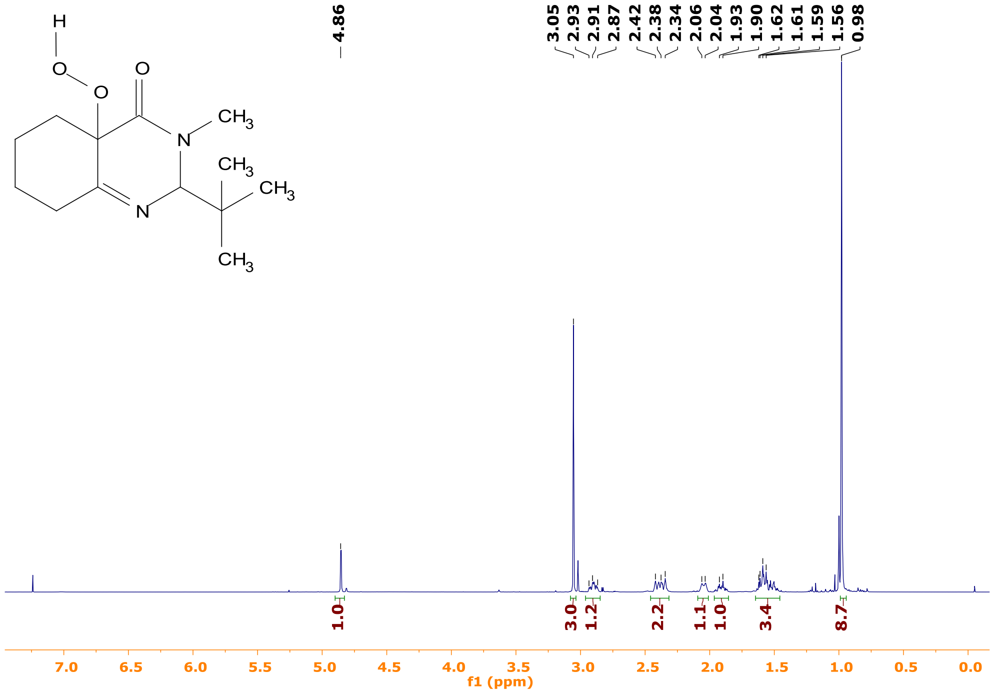

*Figure S5. ^{1}H spectrum (500 MHz, $CDCl_3$) of **12***

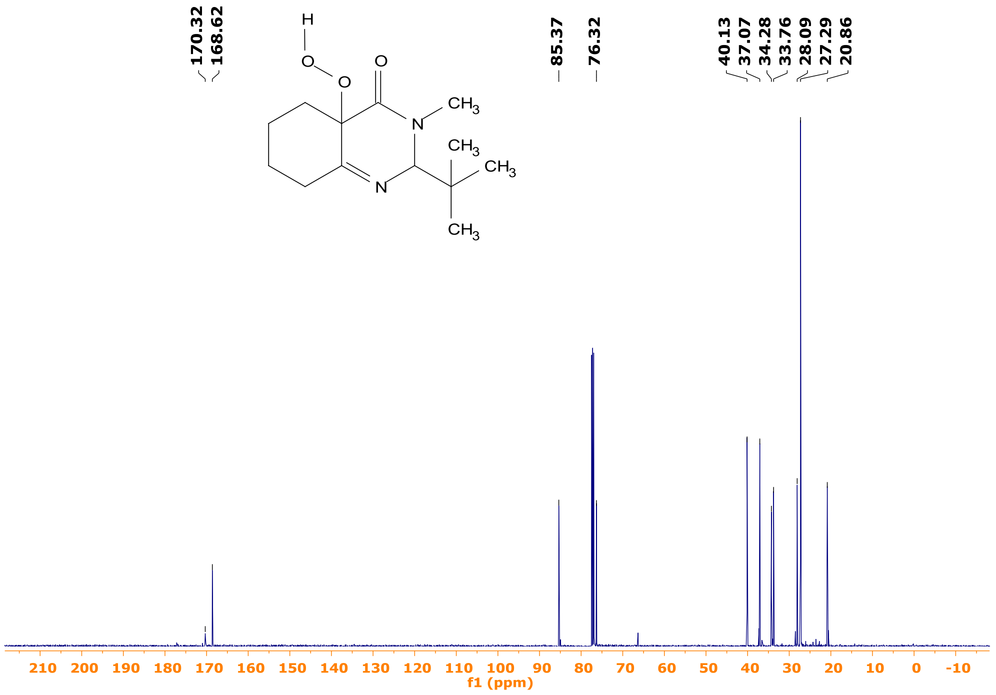

*Figure S6. ^{13}C spectrum (125 MHz, $CDCl_3$) of **12***

9) ^{1}H and ^{13}C NMR spectra for compounds 13.

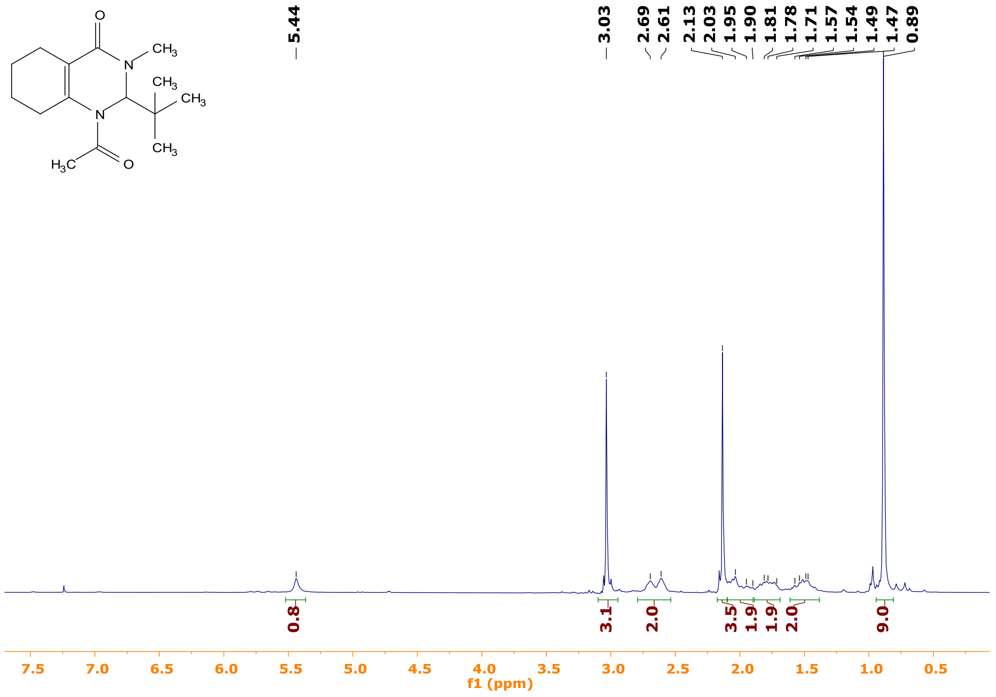

Figure S7. ^{1}H spectrum (200 MHz, $CDCl_3$) of **13**

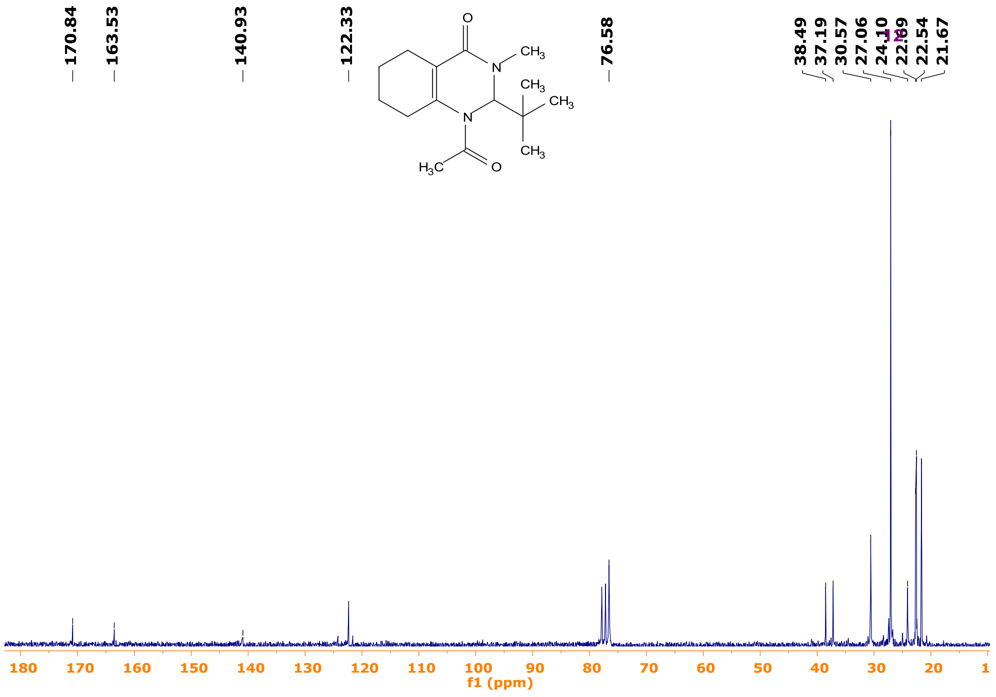

Figure S8. ^{13}C spectrum (50 MHz, $CDCl_3$) of **13**

10) Crystallographic data and structure refinement for product 8.

Table S1. Crystal data and structure refinement for 135leo (**2-(*tert*-butyl)-3-methyl-2,3-dihydroquinazolin-4(*1H*)-one (8)**).

Identification code	135leo
Empirical formula	$C_{33}H_{45}N_3O_7$
Formula weight	595.72
Temperature/K	293(2)
Crystal system	monoclinic
Space group	$P2_1$
a/Å	6.91475(15)
b/Å	11.68447(15)
c/Å	8.08083(14)
α/°	90
β/°	113.833(2)
γ/°	90
Volume/Å^3	597.22(2)
Z	1
ρ_{calc}g/cm^3	1.656
μ/mm^{-1}	0.945
F(000)	320.0
Crystal size/mm^3	0.21 × 0.23 × 0.34
Radiation	CuKα (λ = 1.54184)
2Θ range for data collection/ °	11.974 to 144.606
Index ranges	$-8 \leq h \leq 8$, $-14 \leq k \leq 14$, $-9 \leq l \leq 9$
Reflections collected	7294
Independent reflections	2301 [R_{int} = 0.0152, R_{sigma} = 0.0121]
Data/restraints/parameters	2301/1/149
Goodness-of-fit on F^2	1.094
Final R indexes [I>=2σ (I)]	R_1 = 0.0338, wR_2 = 0.1120
Final R indexes [all data]	R_1 = 0.0339, wR_2 = 0.1122
Largest diff. peak/hole / e Å^{-3}	0.37/-0.30
Flack parameter	0.52(7)

Table S2 Fractional Atomic Coordinates (×10^4) and Equivalent Isotropic Displacement Parameters (Å^2×10^3) for 135leo (2-(*tert*-butyl)-3-methyl-2,3-dihydroquinazolin-4(*1H*)-one (8)). U_{eq} is defined as 1/3 of of the trace of the orthogonalised U_{IJ} tensor.

Atom	*x*	*y*	*z*	U(eq)
O1	-18(2)	4586.0(15)	7283.1(18)	30.2(4)
N1	5666(2)	4418.1(12)	7052.9(18)	18.0(4)
N3	2066(2)	3862.8(12)	5969.7(19)	19.6(4)
C2	3904(2)	3974.0(13)	5491(2)	17.2(4)
C4	1632(3)	4626.8(16)	7025(2)	20.4(4)
C4A	3293(3)	5478.8(14)	7963(2)	18.5(4)
C5	2919(3)	6394.6(15)	8910(2)	22.8(4)
C6	4517(3)	7127.5(16)	9931(3)	26.1(4)
C7	6559(3)	6926.3(15)	10036(2)	27.4(4)
C8	6974(3)	6029.0(16)	9108(2)	22.4(4)
C8A	5322(3)	5308.0(14)	8020(2)	17.3(3)
C9	3444(3)	4641.7(14)	3697(2)	20.2(4)
C10	1528(3)	4110.3(17)	2162(2)	27.0(4)
C11	5388(3)	4510.0(17)	3249(2)	26.8(4)
C12	2992(4)	5906.2(16)	3857(3)	29.9(5)
C13	679(3)	2874.7(17)	5302(3)	29.3(4)

Table S3 Anisotropic Displacement Parameters (Å^2×10^3) for 135leo(2-(*tert*-butyl)-3-methyl-2,3-dihydroquinazolin-4(*1H*)-one (8)). The Anisotropic displacement factor exponent takes the form: $-2\pi^2[h^2a^{*2}U_{11}+2hka^*b^*U_{12}+\ldots]$.

Atom	U_{11}	U_{22}	U_{33}	U_{23}	U_{13}	U_{12}
O1	16.7(6)	48.3(8)	28.8(7)	-4.3(6)	12.4(5)	-7.2(6)
N1	12.5(7)	20.6(7)	22.0(7)	0.4(5)	8.2(6)	2.5(5)
N3	17.6(7)	20.9(7)	20.5(6)	0.1(5)	8.0(5)	-4.9(5)
C2	17.8(7)	13.6(7)	21.5(8)	0.6(5)	9.4(6)	1.4(5)
C4	16.0(8)	27.1(8)	19.8(8)	3.3(6)	9.1(6)	0.6(7)
C4A	18.3(8)	21.7(8)	17.0(7)	2.3(6)	8.6(6)	-0.3(6)
C5	25.7(8)	24.8(9)	22.7(8)	3.1(6)	14.6(7)	3.9(7)
C6	40.2(12)	21.2(8)	22.9(8)	-2.7(6)	19.0(8)	-3.1(7)
C7	34.3(10)	26.4(9)	21.8(8)	-2.8(7)	11.7(7)	-10.7(7)
C8	20.3(8)	26.7(9)	22.4(9)	1.4(6)	10.7(6)	-5.2(7)
C8A	17.2(7)	18.9(7)	17.0(7)	4.3(6)	8.2(6)	0.9(6)
C9	23.8(8)	19.9(8)	20.3(8)	0.4(6)	12.3(6)	2.6(6)
C10	25.7(10)	33.4(10)	20.1(8)	1.5(7)	7.6(7)	5.0(7)
C11	26.1(9)	34.3(10)	24.8(8)	1.6(7)	15.3(7)	1.8(8)
C12	47.9(11)	21.3(9)	26.9(10)	6.3(7)	21.8(9)	9.8(8)
C13	26.1(9)	29.5(10)	31.7(10)	-4.2(7)	11.1(7)	-11.1(7)

Table S4 Bond Lengths for 135leo (2-(*tert*-butyl)-3-methyl-2,3-dihydroquinazolin-4(*1H*)-one (8)).

Atom	Atom	Length/Å	Atom	Atom	Length/Å
O1	C4	1.241(2)	C4A	C8A	1.399(2)
N1	C2	1.4508(19)	C5	C6	1.378(3)
N1	C8A	1.378(2)	C6	C7	1.400(3)
N3	C2	1.476(2)	C7	C8	1.385(3)
N3	C4	1.349(2)	C8	C8A	1.406(2)
N3	C13	1.458(2)	C9	C10	1.534(2)
C2	C9	1.561(2)	C9	C11	1.535(2)
C4	C4A	1.477(2)	C9	C12	1.526(2)
C4A	C5	1.399(2)			

Table S5 Bond Angles for 135leo (2-(*tert*-butyl)-3-methyl-2,3-dihydroquinazolin-4(*1H*)-one (8)).

Atom	Atom	Atom	Angle/°	Atom	Atom	Atom	Angle/°
C8A	N1	C2	119.09(13)	C6	C5	C4A	121.47(17)
C4	N3	C2	122.82(13)	C5	C6	C7	118.52(17)
C4	N3	C13	118.49(15)	C8	C7	C6	121.25(17)
C13	N3	C2	118.68(14)	C7	C8	C8A	119.91(16)
N1	C2	N3	108.25(12)	N1	C8A	C4A	119.33(15)
N1	C2	C9	114.41(13)	N1	C8A	C8	121.58(14)
N3	C2	C9	114.41(12)	C4A	C8A	C8	119.05(15)
O1	C4	N3	122.34(16)	C10	C9	C2	109.52(14)
O1	C4	C4A	121.11(16)	C10	C9	C11	108.71(13)
N3	C4	C4A	116.43(14)	C11	C9	C2	107.45(13)
C5	C4A	C4	121.27(16)	C12	C9	C2	112.17(13)
C5	C4A	C8A	119.71(16)	C12	C9	C10	108.71(15)
C8A	C4A	C4	118.82(15)	C12	C9	C11	110.21(15)

Table S6 Hydrogen Atom Coordinates (Å×10[4]) and Isotropic Displacement Parameters (Å[2]×10[3]) for 135leo (2-(*tert*-butyl)-3-methyl-2,3-dihydroquinazolin-4(*1H*)-one (8)).

Atom	*x*	*y*	*z*	U(eq)
H1	6909	4130	7371	22
H2	4294	3195	5298	21
H5	1561	6511	8849	27
H6	4245	7742	10536	31
H7	7658	7404	10743	33
H8	8344	5903	9205	27
H10A	1700	3295	2167	40
H10B	1418	4415	1024	40
H10C	266	4289	2334	40
H11A	6596	4847	4196	40
H11B	5133	4889	2125	40
H11C	5651	3712	3144	40
H12A	1825	5977	4206	45
H12B	2646	6275	2711	45
H12C	4221	6261	4753	45
H13A	-397	3044	4129	44
H13B	30	2701	6122	44
H13C	1491	2228	5216	44

11) Crystallographic data and structure refinement for product 12.

Table S7. Crystal data and structure refinement for leo144a (**2-(*tert*-butyl)-4a-hydroperoxy-3-methyl-2,4a,5,6,7,8-hexahydroquinazolin-4(*3H*)-one (12)**.

Identification code	leo144a
Empirical formula	$C_{13}H_{22}N_2O_3$
Formula weight	254.32
Temperature/K	100.01(11)
Crystal system	orthorhombic
Space group	$Pna2_1$
a/Å	12.0759(8)
b/Å	9.9245(6)
c/Å	22.5539(16)
α/°	90
β/°	90
γ/°	90
Volume/Å^3	2703.0(3)
Z	8
ρ_{calc}g/cm^3	1.25
μ/mm^{-1}	0.089
F(000)	1104
Crystal size/mm^3	0.4 × 0.3 × 0.3
Radiation	MoKα (λ = 0.71073)
2Θ range for data collection/°	5.312 to 58.264
Index ranges	-16 ≤ h ≤ 8, -13 ≤ k ≤ 13, -28 ≤ l ≤ 26
Reflections collected	10662
Independent reflections	5466 [R_{int} = 0.0280, R_{sigma} = 0.0396]
Data/restraints/parameters	5466/1/341
Goodness-of-fit on F^2	1.041
Final R indexes [I>=2σ (I)]	R_1 = 0.0460, wR_2 = 0.1095
Final R indexes [all data]	R_1 = 0.0532, wR_2 = 0.1136
Largest diff. peak/hole / e Å^{-3}	0.30/-0.25
Flack parameter	-0.3(7)

Table S8 Fractional Atomic Coordinates (×104) and Equivalent Isotropic Displacement Parameters (Å2×103) for leo144a (2-(*tert*-butyl)-4a-hydroperoxy-3-methyl-2,4a,5,6,7,8-hexahydroquinazolin-4(*3H*)-one (12). Ueq is defined as 1/3 of of the trace of the orthogonalised UIJ tensor.

Atom	*x*	*y*	*z*	U(eq)
C2	9027(2)	2972(3)	3835.8(14)	15.6(6)
C4	7266(2)	3998(3)	4178.9(14)	15.9(6)
C4A	7826(2)	5374(3)	4180.2(14)	15.4(6)
C5	7335(3)	6364(3)	3729.5(15)	18.5(7)
C6	7911(3)	7736(4)	3773(2)	21.2(7)
C7	9162(3)	7601(3)	3691(2)	23.0(9)
C8	9655(3)	6610(3)	4143.5(15)	19.8(7)
C8A	9072(2)	5285(3)	4092.9(14)	14.8(6)
C9	9102(3)	2616(3)	3159.5(19)	15.3(7)
C10	8502(3)	3680(4)	2784.7(16)	21.8(8)
C11	8593(3)	1234(4)	3038.3(17)	25.2(8)
C12	10335(3)	2581(4)	2995(2)	23.7(10)
C13	7434(3)	1609(3)	4268.2(17)	21.4(7)
N1	9591(2)	4235(3)	3933.5(12)	15.1(5)
N3	7885(2)	2913(3)	4066.8(12)	14.9(5)
O1	7573.1(19)	6001(2)	4732.6(10)	20.0(5)
O2	8021.2(19)	5146(2)	5194.6(11)	22.4(5)
O4	6276.4(17)	3894(2)	4318.0(11)	21.8(5)
C15	3571(2)	2991(3)	6158.4(14)	15.8(6)
C17	5336(2)	4024(3)	5830.9(13)	15.1(6)
C17A	4769(2)	5393(3)	5823.1(14)	14.9(6)
C18	5249(3)	6402(3)	6273.7(16)	19.8(7)
C19	4660(3)	7761(4)	6235.0(19)	20.5(7)
C20	3408(3)	7610(3)	6299(2)	19.4(8)
C21	2934(2)	6619(3)	5842.0(15)	17.3(6)
C21A	3524(2)	5296(3)	5900.4(14)	15.0(6)
C22	3496(3)	2652(4)	6832(2)	19.2(8)
C23	4112(3)	3684(3)	7207.0(15)	18.8(7)
C24	2268(3)	2635(4)	7012(2)	22.7(9)
C25	3976(3)	1255(4)	6962.4(19)	28.3(9)
C26	5180(3)	1631(3)	5745.1(17)	22.2(7)
N14	3005(2)	4251(3)	6053.0(12)	15.0(5)
N16	4717(2)	2938(3)	5928.5(12)	15.9(6)
O5	5034.2(19)	6030(2)	5271.1(10)	20.0(5)

O6	4598.6(19)	5174(2)	4805.1(11)	24.7(5)
O17	6332.6(17)	3928(2)	5705.2(11)	22.0(5)

Table S9 Anisotropic Displacement Parameters (Å2 ×103) for leo144a (2-(*tert*-butyl)-4a-hydroperoxy-3-methyl-2,4a,5,6,7,8-hexahydroquinazolin-4(*3H*)-one (12). The Anisotropic displacement factor exponent takes the form: -2π2[h2a*2U11+2hka*b*U12+…].

Atom	U_{11}	U_{22}	U_{33}	U_{23}	U_{13}	U_{12}
C2	11.9(14)	16.9(16)	17.9(16)	1.7(12)	1.6(12)	2.2(12)
C4	14.2(14)	19.5(15)	14.1(16)	-4.6(13)	2.2(12)	-2.5(13)
C4A	11.5(13)	16.8(14)	17.9(16)	-2.9(12)	-0.7(12)	1.4(12)
C5	13.8(14)	19.9(15)	21.7(17)	-0.5(13)	0.4(13)	3.4(13)
C6	21.2(17)	17.3(14)	25(2)	-0.9(17)	-0.3(14)	1.7(14)
C7	19.2(17)	18.9(18)	31(3)	1.9(14)	2.9(17)	-1.9(13)
C8	13.4(14)	21.1(16)	24.8(18)	-1.6(14)	0.2(12)	-3.5(13)
C8A	12.7(14)	18.2(15)	13.6(15)	2.6(11)	-3.3(11)	-2.5(13)
C9	14.5(15)	17.2(16)	14.3(19)	-1.0(12)	1.5(13)	1.0(12)
C10	18.9(16)	27.0(19)	20(2)	2.2(15)	-1.3(14)	3.6(14)
C11	29.9(19)	21.3(17)	24(2)	-5.2(14)	4.5(15)	-1.2(15)
C12	16.6(17)	32(2)	23(3)	-3.7(14)	2.3(15)	4.8(14)
C13	19.7(16)	17.6(16)	26.8(19)	4.7(15)	1.3(15)	-2.9(14)
N1	11.8(12)	18.0(13)	15.6(14)	1.7(10)	-1.3(10)	0.1(10)
N3	11.6(12)	15.6(13)	17.3(14)	1.9(11)	1.4(10)	0.1(11)
O1	22.1(12)	19.7(11)	18.4(13)	-2.0(9)	1.9(10)	-0.5(10)
O2	21.5(12)	28.9(12)	16.6(12)	2.1(10)	-0.6(9)	-4.2(10)
O4	12.9(11)	26.3(12)	26.1(13)	-10.5(10)	5.5(9)	-3.7(9)
C15	11.0(14)	15.4(16)	21.0(17)	-1.9(12)	0.2(12)	-1.8(12)
C17	14.8(14)	21.5(16)	8.9(15)	3.0(12)	-2.1(12)	0.3(13)
C17A	12.3(13)	18.5(15)	14.0(15)	4.9(12)	2.0(12)	1.9(12)
C18	13.6(14)	19.2(15)	26.7(18)	1.0(13)	-0.5(13)	-1.6(13)
C19	20.3(17)	16.1(14)	25(2)	-3.6(17)	-3.7(14)	-2.7(14)
C20	20.4(17)	18.0(17)	20(2)	-1.3(13)	0.8(15)	2.7(13)
C21	12.3(14)	20.8(16)	18.8(16)	1.1(13)	-1.7(12)	2.7(13)
C21A	14.0(14)	20.5(15)	10.3(15)	-1.4(11)	-2.1(11)	1.4(13)
C22	14.4(15)	19.7(17)	23(2)	2.0(14)	1.6(14)	-0.2(13)
C23	16.2(15)	24.4(17)	15.8(18)	1.9(13)	0.6(13)	-0.1(13)
C24	15.8(15)	31(2)	21(2)	0.4(14)	2.2(15)	-6.2(15)

C25	27.4(18)	23.4(19)	34(2)	7.8(16)	5.2(17)	1.9(16)
C26	21.4(16)	20.6(17)	24.5(19)	-5.7(15)	2.6(14)	2.6(15)
N14	9.9(12)	18.1(13)	16.8(14)	-2(1)	-0.6(10)	0.7(11)
N16	13.4(13)	16.9(15)	17.4(15)	-0.9(11)	0.1(10)	3.8(11)
O5	24.0(11)	20.6(11)	15.4(11)	3.5(9)	3.8(10)	0.5(10)
O6	21.9(12)	30.6(13)	21.6(13)	-2.4(10)	-0.1(10)	6.5(11)
O17	13.4(11)	24.8(12)	27.9(13)	8.2(10)	6.4(10)	4.1(9)

Table S10 Bond Lengths for leo144a (2-(*tert*-butyl)-4a-hydroperoxy-3-methyl-2,4a,5,6,7,8-hexahydroquinazolin-4(*3H*)-one (12).

Atom	Atom	Length/Å	Atom	Atom	Length/Å
C2	C9	1.568(5)	C15	C22	1.559(6)
C2	N1	1.444(4)	C15	N14	1.445(4)
C2	N3	1.475(4)	C15	N16	1.479(4)
C4	C4A	1.524(4)	C17	C17A	1.522(4)
C4	N3	1.335(4)	C17	N16	1.330(4)
C4	O4	1.240(4)	C17	O17	1.240(4)
C4A	C5	1.533(4)	C17A	C18	1.540(4)
C4A	C8A	1.520(4)	C17A	C21A	1.516(4)
C4A	O1	1.426(4)	C17A	O5	1.433(4)
C5	C6	1.532(5)	C18	C19	1.528(5)
C6	C7	1.528(5)	C19	C20	1.527(5)
C7	C8	1.537(5)	C20	C21	1.536(5)
C8	C8A	1.495(4)	C21	C21A	1.500(4)
C8A	N1	1.269(4)	C21A	N14	1.260(4)
C9	C10	1.534(5)	C22	C23	1.523(5)
C9	C11	1.528(5)	C22	C24	1.538(5)
C9	C12	1.535(5)	C22	C25	1.531(5)
C13	N3	1.476(4)	C26	N16	1.472(4)
O1	O2	1.448(3)	O5	O6	1.450(3)

Table S11 Bond Angles for leo144a (2-(*tert*-butyl)-4a-hydroperoxy-3-methyl-2,4a,5,6,7,8-hexahydroquinazolin-4(*3H*)-one (12).

Atom	Atom	Atom	Angle/°	Atom	Atom	Atom	Angle/°
N1	C2	C9	108.5(3)	N14	C15	C22	108.7(3)
N1	C2	N3	114.9(3)	N14	C15	N16	114.6(3)
N3	C2	C9	112.9(3)	N16	C15	C22	112.9(3)
N3	C4	C4A	118.4(3)	N16	C17	C17A	118.2(2)
O4	C4	C4A	120.1(3)	O17	C17	C17A	120.2(3)
O4	C4	N3	121.4(3)	O17	C17	N16	121.4(3)
C4	C4A	C5	113.7(3)	C17	C17A	C18	113.8(2)
C8A	C4A	C4	112.8(2)	C21A	C17A	C17	112.9(3)
C8A	C4A	C5	109.5(2)	C21A	C17A	C18	109.8(2)
O1	C4A	C4	107.3(2)	O5	C17A	C17	107.7(2)
O1	C4A	C5	102.5(2)	O5	C17A	C18	101.7(2)
O1	C4A	C8A	110.5(2)	O5	C17A	C21A	110.5(2)
C6	C5	C4A	110.6(3)	C19	C18	C17A	111.2(3)
C7	C6	C5	111.3(3)	C20	C19	C18	111.6(3)
C6	C7	C8	111.1(3)	C19	C20	C21	111.6(3)
C8A	C8	C7	109.2(3)	C21A	C21	C20	108.9(3)
C8	C8A	C4A	113.9(3)	C21	C21A	C17A	114.0(3)
N1	C8A	C4A	125.0(3)	N14	C21A	C17A	125.2(3)
N1	C8A	C8	120.7(3)	N14	C21A	C21	120.5(3)
C10	C9	C2	110.7(3)	C23	C22	C15	111.6(3)
C10	C9	C12	109.9(3)	C23	C22	C24	109.4(3)
C11	C9	C2	110.7(3)	C23	C22	C25	108.5(3)
C11	C9	C10	109.2(3)	C24	C22	C15	108.4(3)
C11	C9	C12	109.1(3)	C25	C22	C15	111.2(3)
C12	C9	C2	107.2(3)	C25	C22	C24	107.7(3)
C8A	N1	C2	121.6(2)	C21A	N14	C15	121.4(2)
C2	N3	C13	119.2(3)	C17	N16	C15	123.7(3)
C4	N3	C2	123.9(3)	C17	N16	C26	117.0(3)
C4	N3	C13	116.3(2)	C26	N16	C15	119.0(3)
C4A	O1	O2	107.0(2)	C17A	O5	O6	106.9(2)

Table S12 Torsion Angles for leo144a (2-(*tert*-butyl)-4a-hydroperoxy-3-methyl-2,4a,5,6,7,8-hexahydroquinazolin-4(*3H*)-one (12).

A	B	C	D	Angle/˚	A	B	C	D	Angle/˚
C4	C4A	C5	C6	178.0(3)	C17	C17A	C18	C19	178.9(3)
C4	C4A	C8A	C8	-175.1(3)	C17	C17A	C21A	C21	-175.0(3)
C4	C4A	C8A	N1	11.7(4)	C17	C17A	C21A	N14	11.3(4)
C4	C4A	O1	O2	60.8(3)	C17	C17A	O5	O6	61.2(3)
C4A	C4	N3	C2	-11.8(4)	C17A	C17	N16	C15	-14.0(4)
C4A	C4	N3	C13	159.5(3)	C17A	C17	N16	C26	160.5(3)
C4A	C5	C6	C7	56.1(4)	C17A	C18	C19	C20	54.2(4)
C4A	C8A	N1	C2	-2.1(5)	C17A	C21A	N14	C15	-1.6(5)
C5	C4A	C8A	C8	57.2(3)	C18	C17A	C21A	C21	56.9(3)
C5	C4A	C8A	N1	-116.0(3)	C18	C17A	C21A	N14	-116.8(3)
C5	C4A	O1	O2	-179.1(2)	C18	C17A	O5	O6	-179.0(2)
C5	C6	C7	C8	-56.4(5)	C18	C19	C20	C21	-55.5(5)
C6	C7	C8	C8A	55.6(4)	C19	C20	C21	C21A	55.7(4)
C7	C8	C8A	C4A	-57.1(4)	C20	C21	C21A	C17A	-57.5(4)
C7	C8	C8A	N1	116.4(3)	C20	C21	C21A	N14	116.6(3)
C8	C8A	N1	C2	-174.9(3)	C21	C21A	N14	C15	-174.9(3)
C8A	C4A	C5	C6	-54.9(3)	C21A	C17A	C18	C19	-53.5(4)
C8A	C4A	O1	O2	-62.5(3)	C21A	C17A	O5	O6	-62.4(3)
C9	C2	N1	C8A	113.6(3)	C22	C15	N14	C21A	112.3(3)
C9	C2	N3	C4	-103.7(3)	C22	C15	N16	C17	-101.6(3)
C9	C2	N3	C13	85.3(3)	C22	C15	N16	C26	84.1(3)
N1	C2	C9	C10	-63.0(3)	N14	C15	C22	C23	-64.7(3)
N1	C2	C9	C11	175.7(3)	N14	C15	C22	C24	55.8(3)
N1	C2	C9	C12	56.9(3)	N14	C15	C22	C25	174.1(3)
N1	C2	N3	C4	21.4(4)	N14	C15	N16	C17	23.5(4)
N1	C2	N3	C13	-149.6(3)	N14	C15	N16	C26	-150.9(3)
N3	C2	C9	C10	65.5(3)	N16	C15	C22	C23	63.6(3)
N3	C2	C9	C11	-55.7(4)	N16	C15	C22	C24	-175.9(3)
N3	C2	C9	C12	-174.6(3)	N16	C15	C22	C25	-57.7(4)
N3	C2	N1	C8A	-13.8(4)	N16	C15	N14	C21A	-15.0(4)
N3	C4	C4A	C5	121.2(3)	N16	C17	C17A	C18	123.0(3)
N3	C4	C4A	C8A	-4.3(4)	N16	C17	C17A	C21A	-3.0(4)
N3	C4	C4A	O1	-126.2(3)	N16	C17	C17A	O5	-125.2(3)
O1	C4A	C5	C6	62.4(3)	O5	C17A	C18	C19	63.4(3)
O1	C4A	C8A	C8	-55.0(3)	O5	C17A	C21A	C21	-54.4(3)

O1	C4A	C8A	N1	131.8(3)	O5	C17A	C21A	N14	131.8(3)
O4	C4	C4A	C5	-63.5(4)	O17	C17	C17A	C18	-62.3(4)
O4	C4	C4A	C8A	171.0(3)	O17	C17	C17A	C21A	171.7(3)
O4	C4	C4A	O1	49.1(4)	O17	C17	C17A	O5	49.5(4)
O4	C4	N3	C2	172.9(3)	O17	C17	N16	C15	171.3(3)
O4	C4	N3	C13	-15.8(5)	O17	C17	N16	C26	-14.2(4)

Table S13 Hydrogen Atom Coordinates (Å×104) and Isotropic Displacement Parameters (Å2×103) for leo144a (2-(*tert*-butyl)-4a-hydroperoxy-3-methyl-2,4a,5,6,7,8-hexahydroquinazolin-4(*3H*)-one (12).

Atom	*x*	*y*	*z*	U(eq)
H2	9450	2281	4048	19
H5A	7425	6006	3332	22
H5B	6549	6473	3804	22
H6A	7617	8333	3471	25
H6B	7758	8134	4157	25
H7A	9507	8476	3739	28
H7B	9318	7285	3293	28
H8A	10440	6490	4067	24
H8B	9568	6965	4541	24
H10A	7739	3726	2903	33
H10B	8546	3437	2373	33
H10C	8845	4542	2843	33
H11A	8961	567	3275	38
H11B	8678	1015	2626	38
H11C	7820	1251	3137	38
H12A	10643	3469	3034	35
H12B	10415	2279	2593	35
H12C	10717	1974	3256	35
H13A	7175	1693	4669	32
H13B	8003	935	4250	32
H13C	6830	1350	4016	32
H2A	7320(40)	4840(50)	5330(20)	65(16)
H15	3152	2293	5948	19
H18A	5169	6041	6671	24
H18B	6033	6525	6197	24
H19A	4936	8349	6545	25

H19B	4826	8177	5856	25
H20A	3059	8483	6249	23
H20B	3237	7291	6696	23
H21A	2147	6495	5910	21
H21B	3034	6975	5445	21
H23A	4877	3706	7091	28
H23B	4060	3439	7618	28
H23C	3789	4558	7149	28
H24A	1976	3534	6995	34
H24B	2199	2293	7409	34
H24C	1861	2067	6745	34
H25A	3659	609	6695	42
H25B	3804	1005	7363	42
H25C	4764	1275	6911	42
H26A	5393	1674	5335	33
H26B	4632	940	5797	33
H26C	5817	1427	5983	33
H6	5260(40)	4800(40)	4630(20)	44(13)

12) Crystallographic data and structure refinement for product 13.

Table S14. Crystal data and structure refinement for redn (**1-acetyl-2-(*tert*-butyl)-3-methyl-2,3,5,6,7,8-hexahydroquinazolin-4(*1H*)-one (13)**.

Identification code	redn
Empirical formula	$C_{15}H_{24}N_2O_2$
Formula weight	264.36
Temperature/K	99.9(4)
Crystal system	orthorhombic
Space group	Pbca
a/Å	14.0614(2)
b/Å	7.94150(10)
c/Å	25.2493(3)
α/°	90
β/°	90
γ/°	90
Volume/Å^3	2819.55(6)
Z	8
ρ_{calc}g/cm^3	1.246
μ/mm^{-1}	0.658
F(000)	1152
Crystal size/mm^3	0.12 × 0.099 × 0.05
Radiation	CuKα (λ = 1.54184)
2Θ range for data collec	9.414 to 145.654
Index ranges	-17 ≤ h ≤ 16, -9 ≤ k ≤ 6, -31 ≤ l ≤ 31
Reflections collected	18083
Independent reflections	2782 [R_{int} = 0.0248, R_{sigma} = 0.0140]
Data/restraints/paramet	2782/0/177
Goodness-of-fit on F^2	1.072
Final R indexes [I>=2σ (I)]	R_1 = 0.0373, wR_2 = 0.0925
Final R indexes [all data]	R_1 = 0.0391, wR_2 = 0.0939
Largest diff. peak/hole / e Å^{-3}	0.24/-0.18

Table S15 Fractional Atomic Coordinates (×104) and Equivalent Isotropic Displacement Parameters (Å2×103) for redn (1-acetyl-2-(*tert*-butyl)-3-methyl-2,3,5,6,7,8-hexahydroquinazolin-4(*1H*)-one (13). Ueq is defined as 1/3 of of the trace of the orthogonalised UIJ tensor.

Atom	*x*	*y*	*z*	U(eq)
N1	5957.4(7)	2747.8(12)	4006.1(4)	15.4(2)
C2	5098.2(8)	3756.3(14)	4091.2(4)	15.3(2)
N3	5161.5(7)	5282.0(12)	3762.3(4)	16.6(2)
C4	5654.3(8)	5314.1(14)	3300.1(4)	15.7(2)
O4	5779.5(6)	6632.1(10)	3050.7(3)	21.2(2)
C4A	6040.3(8)	3675.4(14)	3114.1(4)	14.8(2)
C5	6207.1(8)	3500.6(14)	2527.9(5)	17.9(2)
C6	6741.7(9)	1891.1(15)	2389.8(5)	22.3(3)
C7	6356.7(9)	429.1(15)	2710.2(5)	20.7(3)
C8	6539.9(9)	712.8(15)	3297.7(5)	19.0(3)
C8A	6213.2(8)	2446.0(14)	3464.2(4)	14.7(2)
C9	6551.9(9)	2490.3(15)	4433.0(5)	19.1(3)
O9	6311.9(7)	2866.4(12)	4882.2(3)	26.9(2)
C10	7519.9(9)	1743.8(18)	4329.3(5)	26.7(3)
C11	4164.3(8)	2726.4(15)	4033.4(5)	17.0(2)
C12	3315.9(9)	3900.0(16)	4134.0(5)	23.8(3)
C13	4180.5(9)	1358.9(16)	4464.1(5)	21.8(3)
C14	4039.0(9)	1892.8(17)	3491.1(5)	22.1(3)
C15	4852.2(9)	6897.5(15)	3983.0(5)	20.9(3)

Table S16 Anisotropic Displacement Parameters (Å2×103) for redn (1-acetyl-2-(*tert*-butyl)-3-methyl-2,3,5,6,7,8-hexahydroquinazolin-4(*1H*)-one (13). The Anisotropic displacement factor exponent takes the form: -2π2[h2a*2U11+2hka*b*U12+…].

Atom	U_{11}	U_{22}	U_{33}	U_{23}	U_{13}	U_{12}
N1	14.8(5)	14.9(5)	16.4(5)	0.2(4)	-0.5(4)	0.4(4)
C2	15.9(5)	14.4(5)	15.6(5)	-0.2(4)	1.0(4)	0.3(4)
N3	18.6(5)	11.6(5)	19.4(5)	-0.2(4)	2.6(4)	0.8(4)
C4	13.2(5)	15.0(5)	18.7(5)	0.4(4)	-1.7(4)	-0.2(4)
O4	23.7(4)	14.2(4)	25.5(4)	4.6(3)	3.5(3)	1.0(3)
C4A	11.9(5)	14.0(5)	18.4(5)	-0.9(4)	0.3(4)	-1.4(4)
C5	18.1(6)	18.0(6)	17.6(5)	1.0(4)	1.5(4)	0.3(5)
C6	24.7(6)	21.6(6)	20.6(6)	-3.7(5)	4.0(5)	0.8(5)

C7	20.8(6)	15.4(6)	25.8(6)	-4.8(5)	2.8(5)	-1.1(5)
C8	18.7(6)	14.3(5)	24.1(6)	0.1(4)	0.9(5)	2.2(4)
C8A	11.9(5)	14.6(5)	17.5(5)	-0.8(4)	0.1(4)	-1.0(4)
C9	21.0(6)	16.6(5)	19.7(6)	3.3(4)	-2.9(4)	-2.9(5)
O9	29.2(5)	34.6(5)	17.0(4)	1.0(4)	-3.5(3)	1.2(4)
C10	22.0(6)	31.7(7)	26.4(6)	2.4(5)	-7.7(5)	5.0(5)
C11	15.2(5)	17.5(5)	18.5(5)	0.0(4)	1.8(4)	-1.0(4)
C12	16.8(6)	23.9(6)	30.6(7)	0.9(5)	4.2(5)	1.0(5)
C13	22.7(6)	20.4(6)	22.4(6)	2.4(5)	2.8(5)	-3.6(5)
C14	17.3(6)	27.7(6)	21.4(6)	-2.6(5)	-0.2(4)	-5.7(5)
C15	22.8(6)	14.5(5)	25.4(6)	-3.3(5)	3.5(5)	2.2(5)

Table S17 Bond Lengths for redn (1-acetyl-2-(*tert*-butyl)-3-methyl-2,3,5,6,7,8-hexahydroquinazolin-4(*1H*)-one (13).

Atom	Atom	Length/Å	Atom	Atom	Length/Å
N1	C2	1.4654(14)	C4A	C8A	1.3393(16)
N1	C8A	1.4348(14)	C5	C6	1.5233(16)
N1	C9	1.3794(15)	C6	C7	1.5151(17)
C2	N3	1.4716(14)	C7	C8	1.5222(16)
C2	C11	1.5539(15)	C8	C8A	1.5107(16)
N3	C4	1.3574(15)	C9	O9	1.2202(15)
N3	C15	1.4649(14)	C9	C10	1.5075(18)
C4	O4	1.2342(14)	C11	C12	1.5351(16)
C4	C4A	1.4862(15)	C11	C13	1.5370(16)
C4A	C5	1.5051(15)	C11	C14	1.5310(16)

Table S18 Bond Angles for redn (1-acetyl-2-(*tert*-butyl)-3-methyl-2,3,5,6,7,8-hexahydroquinazolin-4(*1H*)-one (13).

Atom	Atom	Atom	Angle/°	Atom	Atom	Atom	Angle/°
C8A	N1	C2	115.96(9)	C7	C6	C5	110.15(10)
C9	N1	C2	117.78(10)	C6	C7	C8	110.27(10)
C9	N1	C8A	124.65(10)	C8A	C8	C7	110.77(10)
N1	C2	N3	108.50(9)	N1	C8A	C8	119.59(10)
N1	C2	C11	113.29(9)	C4A	C8A	N1	117.52(10)
N3	C2	C11	115.57(9)	C4A	C8A	C8	122.39(10)
C4	N3	C2	122.10(9)	N1	C9	C10	118.01(10)
C4	N3	C15	117.53(9)	O9	C9	N1	121.50(11)
C15	N3	C2	119.24(9)	O9	C9	C10	120.49(11)
N3	C4	C4A	116.21(10)	C12	C11	C2	108.76(9)
O4	C4	N3	121.83(10)	C12	C11	C13	108.87(10)
O4	C4	C4A	121.95(10)	C13	C11	C2	107.04(9)
C4	C4A	C5	116.65(10)	C14	C11	C2	114.13(9)
C8A	C4A	C4	119.74(10)	C14	C11	C12	108.72(10)
C8A	C4A	C5	123.61(10)	C14	C11	C13	109.20(10)
C4A	C5	C6	112.30(10)				

Table S19 Hydrogen Atom Coordinates (Å×104) and Isotropic Displacement Parameters (Å2×103) for redn (1-acetyl-2-(*tert*-butyl)-3-methyl-2,3,5,6,7,8-hexahydroquinazolin-4(*1H*)-one (13).

**Atom**	***x***	***y***	***z***	**U(eq)**
H2	5122	4137	4460	18
H5A	6569	4462	2403	21
H5B	5599	3503	2346	21
H6A	6671	1654	2015	27
H6B	7413	2037	2464	27
H7A	6662	-606	2598	25
H7B	5679	318	2649	25
H8A	6203	-132	3502	23
H8B	7214	594	3370	23
H10A	7928	1963	4626	40
H10B	7787	2243	4016	40
H10C	7461	550	4279	40
H12A	3401	4470	4466	36
H12B	2741	3250	4145	36
H12C	3275	4714	3854	36
H13A	4699	600	4399	33
H13B	3592	746	4458	33
H13C	4260	1876	4805	33
H14A	4077	2734	3219	33
H14B	3430	1350	3476	33
H14C	4531	1072	3439	33
H15A	5397	7598	4046	31
H15B	4523	6707	4311	31
H15C	4434	7446	3737	31

13) Cartesian coordinates from optimized structures of 11.

Figure S9. Cartesian coordinates from optimized structure of **11** at the B3LYP/6-311++G** level of theory.

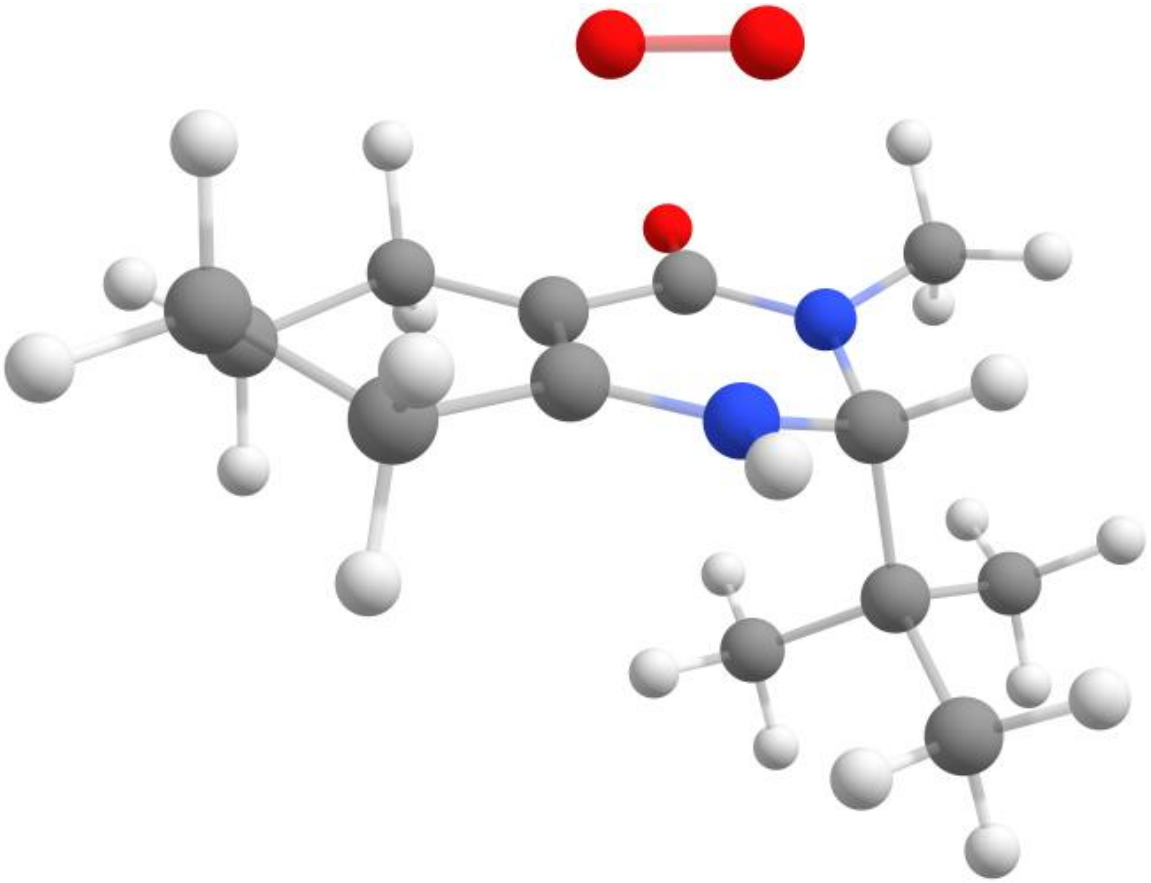

E = -843.603591 Hartree

Negative eigenvalues = 0

6	-3.383965000	-1.351790000	0.093141000
1	-3.797238000	-0.612645000	0.785358000
1	-4.047160000	-2.221289000	0.108021000
6	-3.305495000	-0.746630000	-1.312375000
1	-4.306980000	-0.507085000	-1.680223000
1	-2.885899000	-1.486997000	-2.005259000
6	-1.989583000	-1.766570000	0.579246000
1	-1.685590000	-2.705395000	0.096612000
1	-1.997401000	-1.957657000	1.658626000
6	-2.439128000	0.518106000	-1.315417000
1	-2.989513000	1.357275000	-0.871498000

1	-2.201657000	0.837153000	-2.334376000
6	-1.162433000	0.350530000	-0.544534000
6	-0.941393000	-0.725825000	0.285779000
6	-0.129707000	1.398834000	-0.685448000
7	1.044429000	1.209641000	0.014654000
7	0.219352000	-0.808909000	0.963617000
1	0.296098000	-1.489220000	1.702957000
6	1.427083000	-0.063498000	0.612968000
1	1.936757000	0.162124000	1.554738000
6	1.879442000	2.392315000	0.223576000
1	1.239390000	3.239611000	0.471194000
1	2.456556000	2.655614000	-0.666552000
1	2.564514000	2.203297000	1.049845000
8	-0.330667000	2.424746000	-1.326120000
6	2.421082000	-0.936230000	-0.244253000
6	3.721866000	-0.145280000	-0.477308000
1	3.558579000	0.727431000	-1.110826000
1	4.455519000	-0.782580000	-0.978615000
1	4.167864000	0.191357000	0.463982000
6	1.816411000	-1.326250000	-1.602172000
1	1.555241000	-0.444572000	-2.191862000
1	0.920642000	-1.941210000	-1.488329000
1	2.542846000	-1.906911000	-2.178339000
6	2.764444000	-2.208060000	0.558105000
1	3.155790000	-1.964216000	1.552169000
1	3.534447000	-2.782935000	0.037156000
1	1.899323000	-2.865313000	0.679521000
8	-0.846917000	1.019917000	2.441588000
8	-1.733710000	1.199230000	1.568276000

14) Cartesian coordinates from optimized structures of 12.

Figure S10. Cartesian coordinates from optimized structure of **12** at the B3LYP/6-311++G** level of theory.

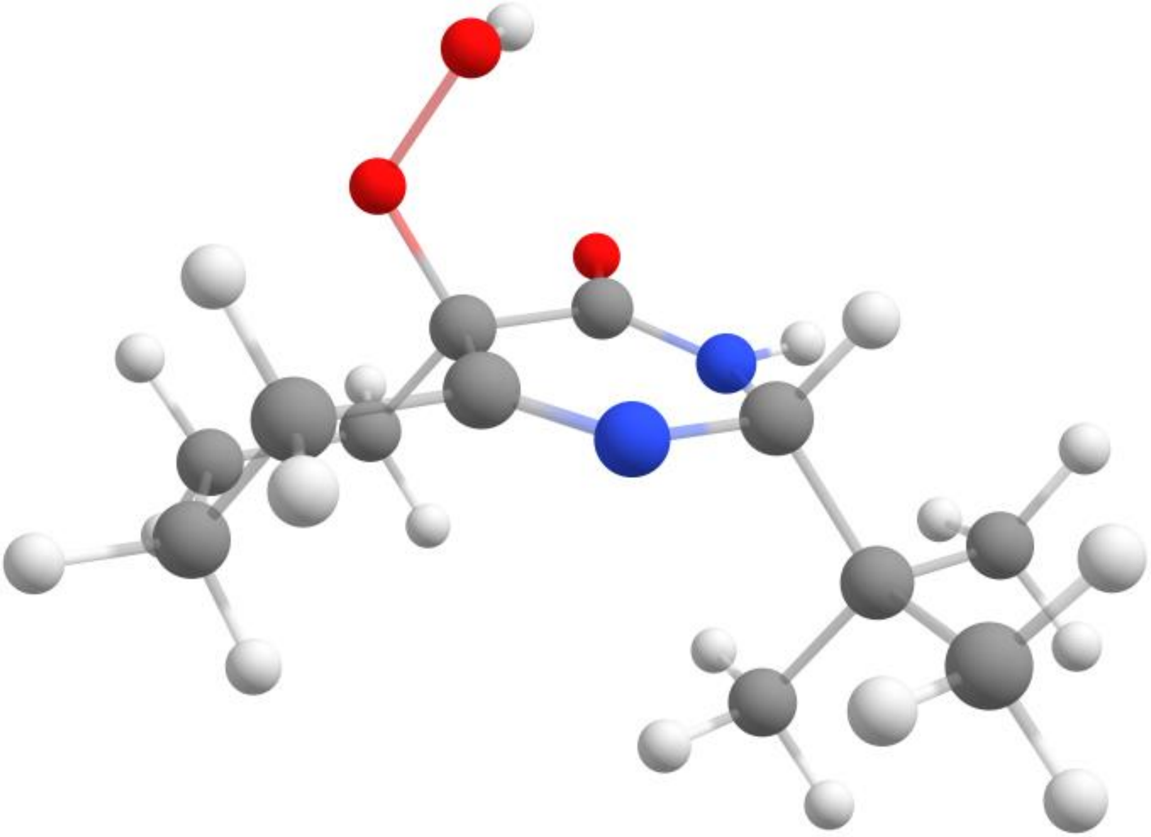

E = -804.377436 Hartree

Negative eigenvalues = 0

6	-2.201070000	-2.399444000	-0.248377000
1	-2.922574000	-3.175300000	0.023628000
1	-1.411408000	-2.893278000	-0.827688000
6	-2.878188000	-1.324618000	-1.110181000
1	-3.762677000	-0.941350000	-0.593732000
1	-3.225597000	-1.761229000	-2.051693000
6	-1.929307000	-0.156814000	-1.423070000
1	-2.438742000	0.645829000	-1.960610000
1	-1.100841000	-0.502177000	-2.050983000
6	-1.579722000	-1.799480000	1.032073000
1	-1.016104000	-2.547638000	1.590796000

1	-2.377642000	-1.414428000	1.675245000
6	-0.653860000	-0.666062000	0.669999000
6	-1.320318000	0.447196000	-0.137135000
8	-2.459898000	0.965723000	0.564540000
8	-2.001126000	1.708626000	1.722762000
1	-2.038728000	2.613623000	1.372988000
7	0.580818000	-0.729596000	0.961122000
6	1.513397000	0.302850000	0.598055000
1	1.839345000	0.759933000	1.543767000
7	0.939569000	1.373754000	-0.235648000
1	1.529445000	2.174171000	-0.426445000
6	-0.374685000	1.606079000	-0.467094000
6	2.794791000	-0.314301000	-0.058106000
6	3.446829000	-1.267235000	0.960214000
1	3.728811000	-0.735928000	1.875116000
1	4.355920000	-1.702276000	0.535282000
1	2.769241000	-2.075193000	1.236332000
6	3.793741000	0.811185000	-0.390440000
1	4.004734000	1.434435000	0.485448000
1	3.442368000	1.456597000	-1.200431000
1	4.742688000	0.379325000	-0.718754000
6	2.432195000	-1.080921000	-1.339862000
1	1.938729000	-0.430519000	-2.067373000
1	1.769601000	-1.921745000	-1.121927000
1	3.336168000	-1.479975000	-1.808553000
8	-0.797509000	2.657827000	-0.925451000

15) Cartesian coordinates from optimized structures of R.

Figure S11. Cartesian coordinates from optimized structure of the **R** at the B3LYP/6-311++G** level of theory.

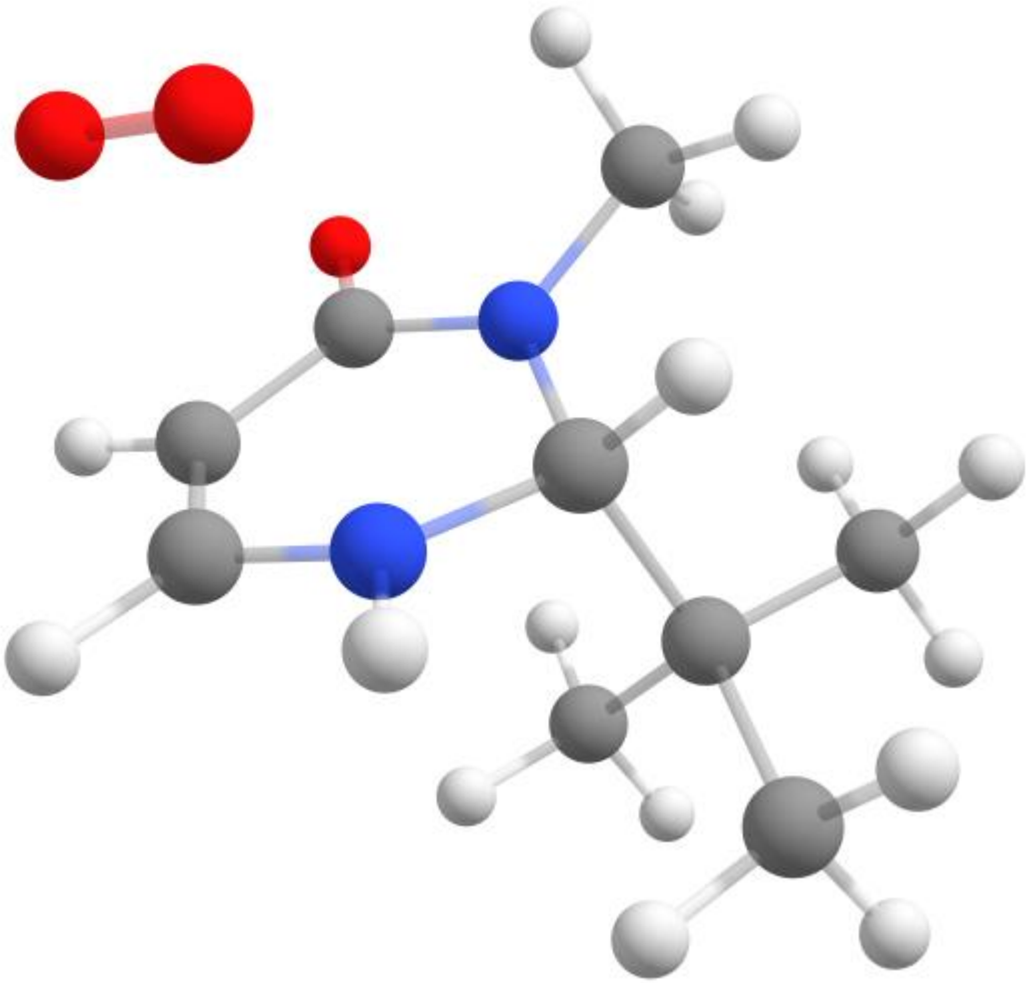

E = -687.587349

Negative eigenvalues = 0

7	0.130729000	-1.451624000	-0.061054000
6	-0.562675000	-0.253416000	-0.543368000
7	0.277861000	0.917533000	-0.300628000
6	1.189945000	1.008707000	0.737606000
6	1.512414000	-0.254859000	1.414939000
6	0.969886000	-1.427424000	0.991019000
1	-0.160675000	-2.338455000	-0.437606000

8	2.945134000	-0.879311000	-0.554593000
8	2.195573000	-1.181577000	-1.502413000
1	1.274407000	-2.386365000	1.392351000
1	2.211754000	-0.215453000	2.235831000
8	1.748978000	2.063862000	1.007528000
6	0.251526000	2.033539000	-1.246021000
1	1.274226000	2.356455000	-1.442712000
1	-0.208018000	1.701576000	-2.176966000
1	-0.303273000	2.892469000	-0.859823000
6	-2.027893000	-0.161660000	0.028218000
6	-2.041444000	0.021307000	1.554029000
1	-1.522002000	0.934488000	1.853522000
1	-3.073715000	0.096831000	1.907890000
1	-1.577000000	-0.820982000	2.071652000
6	-2.751723000	1.026757000	-0.632886000
1	-2.312633000	1.982029000	-0.343377000
1	-2.733917000	0.956415000	-1.725223000
1	-3.799667000	1.038324000	-0.321326000
6	-2.779432000	-1.455060000	-0.348796000
1	-3.833340000	-1.368454000	-0.072537000
1	-2.739896000	-1.646128000	-1.427041000
1	-2.384051000	-2.330975000	0.172704000
1	-0.653419000	-0.364280000	-1.628187000

16) Cartesian coordinates from optimized structures of P.

Figure S12. Cartesian coordinates from optimized structure of the **P** at the B3LYP/6-311++G** level of theory.

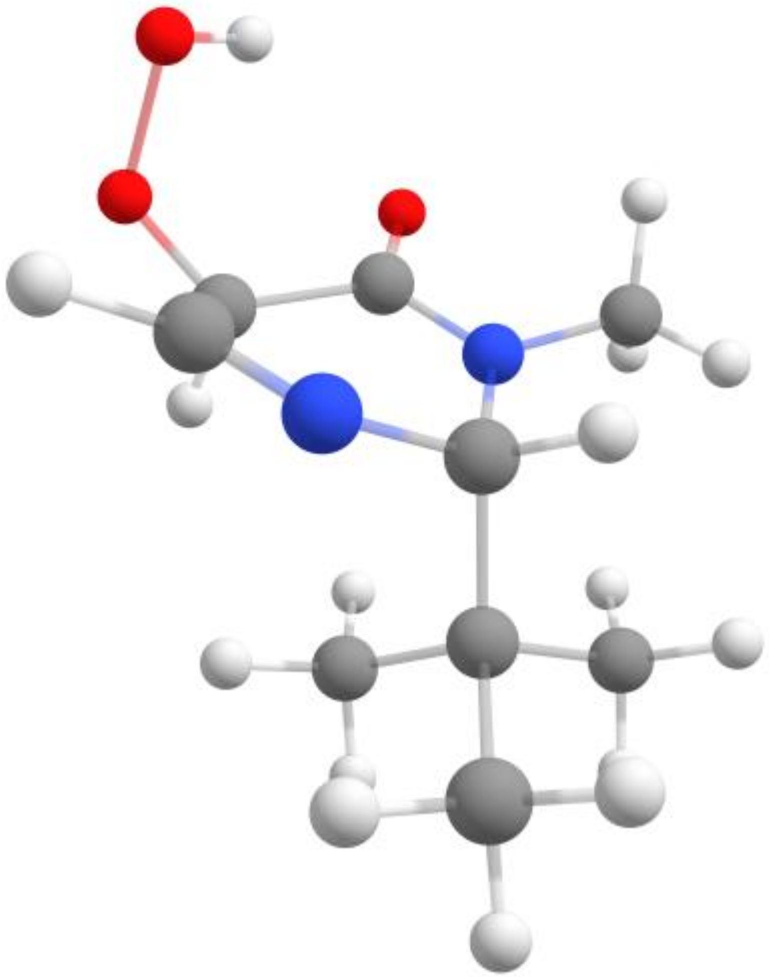

E = -687.652243

Negative eigenvalues = 0

7	-0.270639000	-1.229657000	-1.112563000
6	-0.927317000	0.012062000	-0.748702000
7	-0.000457000	1.054337000	-0.235452000
6	1.211994000	0.769684000	0.302443000
6	1.500956000	-0.719986000	0.512377000
6	0.826687000	-1.540062000	-0.565064000
1	3.681421000	0.438666000	-0.286780000
8	2.880751000	-0.983825000	0.648879000
8	3.560445000	-0.495673000	-0.536340000

1	1.324338000	-2.466730000	-0.852754000
1	1.100183000	-1.025843000	1.486674000
8	2.055374000	1.621322000	0.560907000
6	-0.224527000	2.458513000	-0.604987000
1	0.621711000	2.831061000	-1.185857000
1	-1.135089000	2.533783000	-1.194588000
1	-0.323240000	3.083145000	0.284635000
6	-2.173441000	-0.271195000	0.170079000
6	-1.757575000	-0.871574000	1.523051000
1	-1.111059000	-0.191166000	2.084258000
1	-2.646121000	-1.050925000	2.134413000
1	-1.248556000	-1.830662000	1.402008000
6	-2.941646000	1.039305000	0.422300000
1	-2.347295000	1.762905000	0.984023000
1	-3.263641000	1.506673000	-0.513339000
1	-3.840741000	0.828235000	1.007594000
6	-3.102874000	-1.253346000	-0.570223000
1	-4.016290000	-1.408285000	0.011069000
1	-3.394198000	-0.861102000	-1.549596000
1	-2.623923000	-2.219863000	-0.728747000
1	-1.325934000	0.422341000	-1.682126000

17) Cartesian coordinates from optimized structures of TS1$_Z$.

Figure S13. Cartesian coordinates from optimized structure of the **TS1$_Z$** at the B3LYP/6-311++G** level of theory.

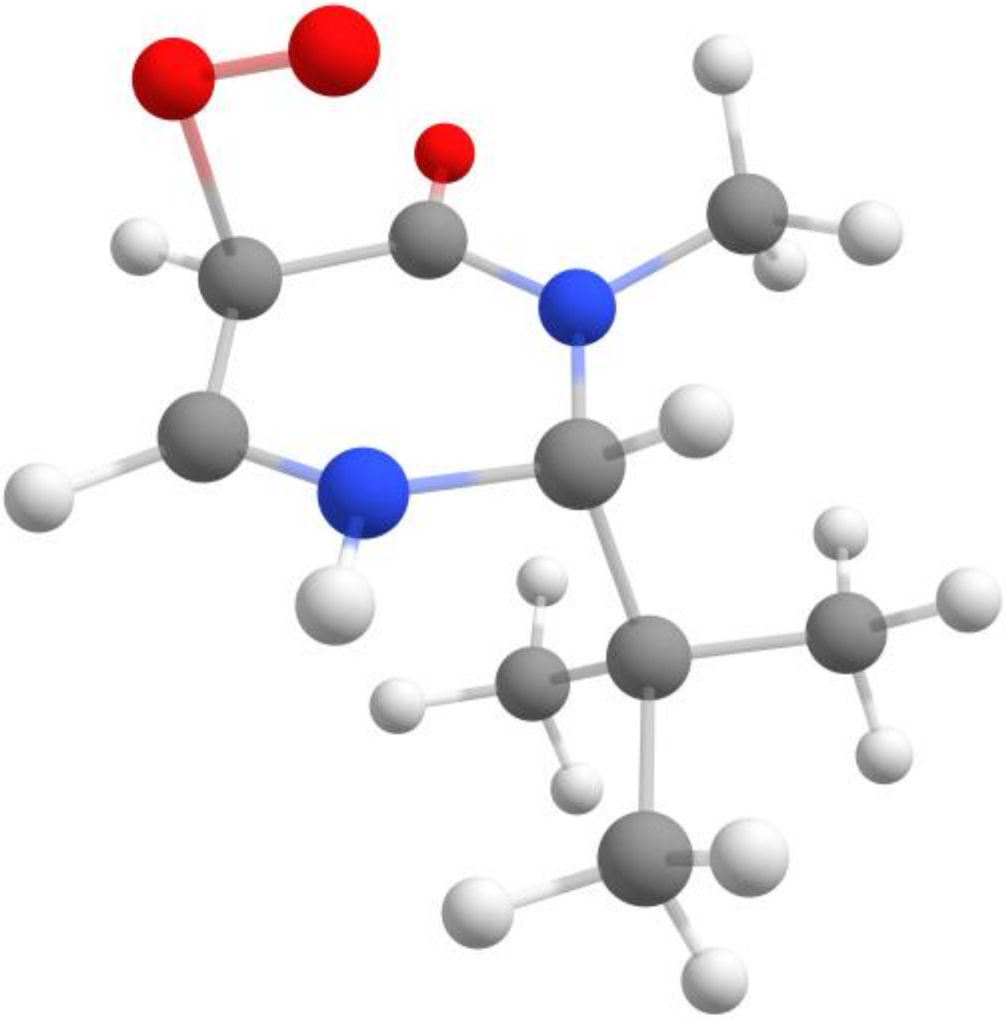

E = -687.577821

Negative eigenvalues = 1; Frequency = -316.1042

7	0.073504000	-1.450200000	-0.233897000
6	-0.589117000	-0.183303000	-0.562414000
7	0.261252000	0.947002000	-0.197269000
6	1.337390000	0.877372000	0.655616000
6	1.811784000	-0.494273000	0.981909000
6	0.968030000	-1.599016000	0.735019000
1	-0.329898000	-2.272900000	-0.655222000

8	2.758492000	-0.935412000	-0.403268000
8	2.134939000	-0.760363000	-1.547936000
1	1.213773000	-2.599540000	1.062934000
1	2.536488000	-0.546380000	1.781852000
8	1.940739000	1.872777000	1.026466000
6	0.104343000	2.211555000	-0.922371000
1	1.093205000	2.581067000	-1.193403000
1	-0.475784000	2.035863000	-1.827012000
1	-0.390157000	2.975356000	-0.317935000
6	-2.037451000	-0.149276000	0.071767000
6	-1.974303000	-0.080545000	1.605538000
1	-1.461440000	0.822849000	1.943379000
1	-2.986571000	-0.063278000	2.019086000
1	-1.459424000	-0.944383000	2.033535000
6	-2.814877000	1.069568000	-0.461710000
1	-2.395889000	2.011602000	-0.109024000
1	-2.839385000	1.089726000	-1.555734000
1	-3.849492000	1.021404000	-0.111548000
6	-2.796871000	-1.420171000	-0.366674000
1	-3.845907000	-1.346087000	-0.070185000
1	-2.776262000	-1.550348000	-1.454596000
1	-2.400752000	-2.326125000	0.100141000
1	-0.702728000	-0.173927000	-1.650290000

18) Cartesian coordinates from optimized structures of $TS1_D$.

Figure S14. Cartesian coordinates from optimized structure of the **$TS1_D$** at the B3LYP/6-311++G** level of theory.

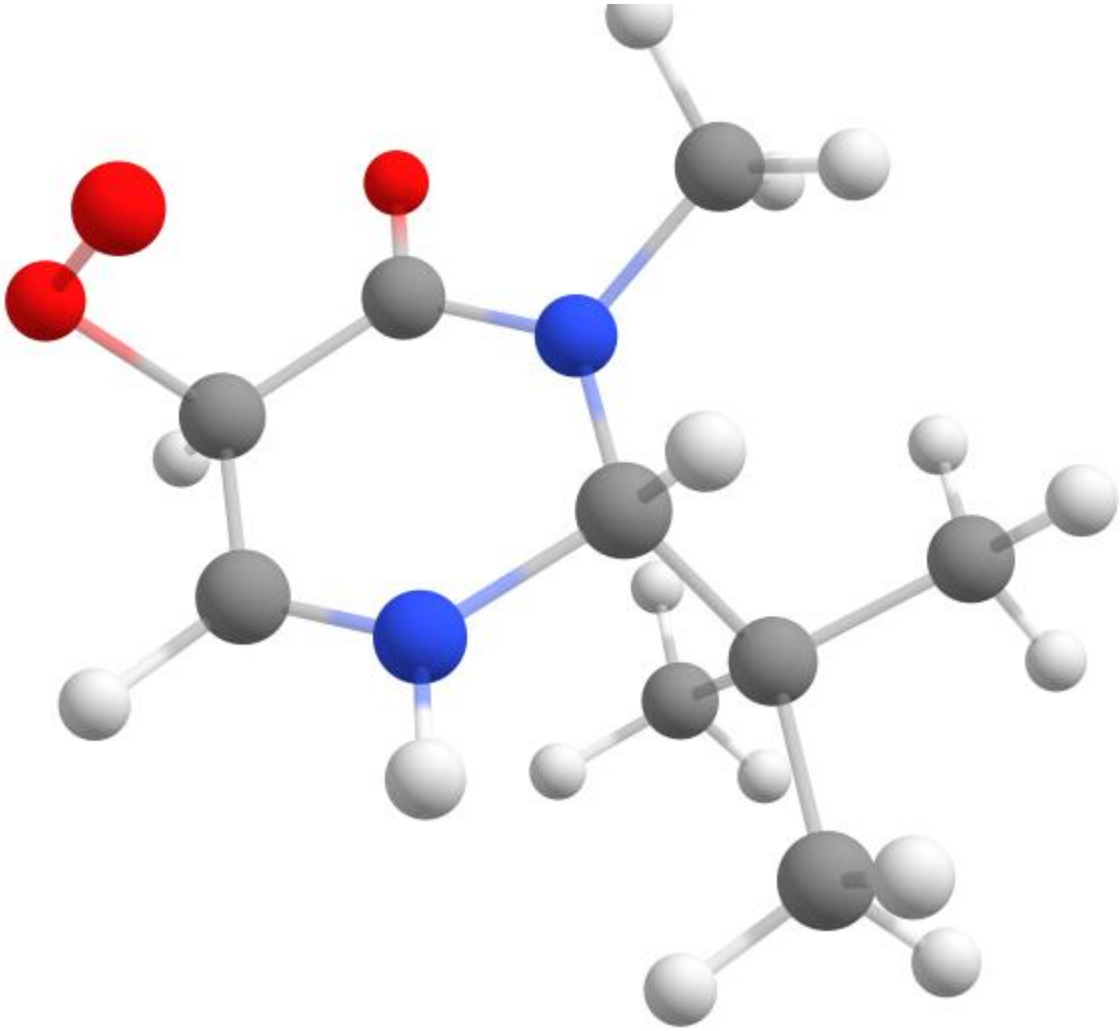

E = -687.562242

Negative eigenvalues = 1; Frequency = -699.8644

7	-0.037849000	-1.446400000	-0.314805000
6	-0.683812000	-0.148251000	-0.570267000
7	0.155188000	0.958062000	-0.151069000
6	1.351516000	0.856437000	0.535939000
6	1.922060000	-0.539583000	0.803839000
6	1.037154000	-1.644254000	0.357474000
1	-0.468781000	-2.234273000	-0.785036000

8	3.029881000	-0.799701000	-0.004437000
8	2.512816000	-0.641302000	-1.403433000
1	1.405647000	-2.660742000	0.447181000
1	2.211511000	-0.630667000	1.856686000
8	1.964563000	1.842360000	0.885344000
6	-0.053487000	2.256724000	-0.813089000
1	0.909366000	2.616038000	-1.175995000
1	-0.731899000	2.130772000	-1.655196000
1	-0.464277000	3.000700000	-0.128015000
6	-2.123500000	-0.186740000	0.085174000
6	-2.015320000	-0.232267000	1.617318000
1	-1.484439000	0.640873000	2.002442000
1	-3.014278000	-0.240320000	2.060975000
1	-1.497756000	-1.129546000	1.968339000
6	-2.925529000	1.059100000	-0.334660000
1	-2.503452000	1.976961000	0.072664000
1	-2.986063000	1.157300000	-1.422619000
1	-3.947040000	0.971989000	0.044154000
6	-2.879756000	-1.430488000	-0.432554000
1	-3.925743000	-1.381079000	-0.122192000
1	-2.871196000	-1.485935000	-1.526945000
1	-2.482245000	-2.367271000	-0.030589000
1	-0.804767000	-0.101372000	-1.659584000

19) Cartesian coordinates from optimized structures of TS2$_Z$.

Figure S15. Cartesian coordinates from optimized structure of the **TS2$_Z$** at the B3LYP/6-311++G** level of theory.

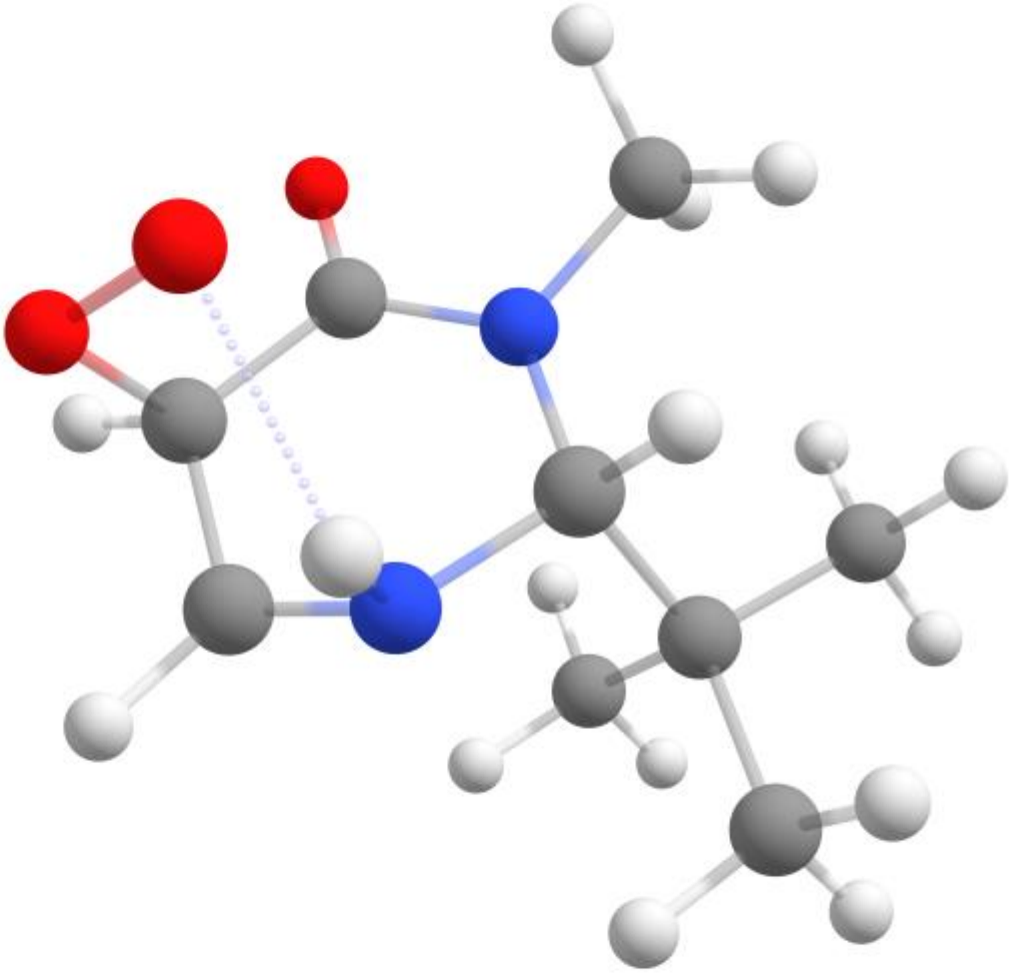

E = -687.571828

Negative eigenvalues = 1; Frequency = -376.1369

7	0.083399000	-1.438665000	-0.324917000
6	-0.589170000	-0.156711000	-0.577114000
7	0.222987000	1.016211000	-0.198152000
6	1.354396000	0.943371000	0.554784000
6	1.830265000	-0.447221000	0.903209000
6	0.898319000	-1.562827000	0.730299000
1	0.597265000	-1.764895000	-1.195158000
8	2.665327000	-1.026165000	-0.222487000

8	2.158838000	-0.846422000	-1.471410000
1	1.199347000	-2.556738000	1.041305000
1	2.487045000	-0.453439000	1.766237000
8	2.012682000	1.918986000	0.880385000
6	-0.017702000	2.303141000	-0.859244000
1	0.938387000	2.707893000	-1.192833000
1	-0.668350000	2.150641000	-1.718399000
1	-0.476319000	3.030190000	-0.185713000
6	-2.018959000	-0.192552000	0.087341000
6	-1.921831000	-0.235185000	1.620895000
1	-1.393540000	0.637130000	2.014413000
1	-2.925738000	-0.238294000	2.054807000
1	-1.412484000	-1.134869000	1.975589000
6	-2.839849000	1.045120000	-0.326461000
1	-2.445843000	1.969152000	0.097068000
1	-2.889887000	1.154788000	-1.414088000
1	-3.864856000	0.933167000	0.036539000
6	-2.759327000	-1.443454000	-0.428591000
1	-3.783384000	-1.450441000	-0.045577000
1	-2.816470000	-1.448868000	-1.522237000
1	-2.269766000	-2.365184000	-0.113316000
1	-0.731744000	-0.106340000	-1.659783000

20) **Cartesian coordinates from optimized structures of $TS2_D$.**

Figure S16. Cartesian coordinates from optimized structure of the **$TS2_D$** at the B3LYP/6-311++G** level of theory.

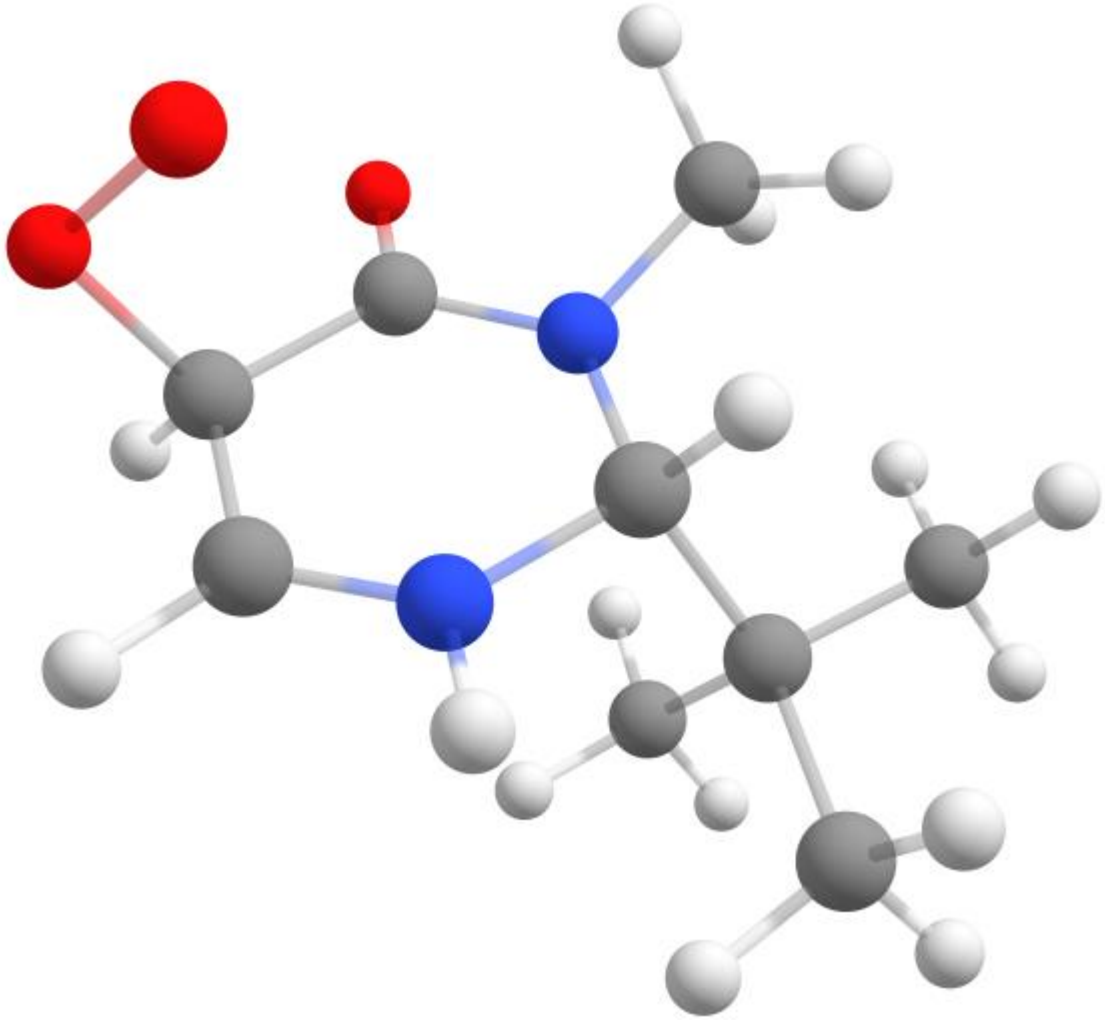

E = -687.562236

Negative eigenvalues = 1; Frequency = -699.7724

7	-0.037869000	-1.446462000	-0.314575000
6	-0.683767000	-0.148288000	-0.570162000
7	0.155162000	0.958041000	-0.150895000
6	1.351677000	0.856400000	0.535820000
6	1.922216000	-0.539621000	0.803722000
6	1.037243000	-1.644310000	0.357528000

1	-0.468944000	-2.234312000	-0.784695000
8	3.030061000	-0.799567000	-0.004562000
8	2.512758000	-0.641206000	-1.403564000
1	1.405457000	-2.660860000	0.447646000
1	2.211650000	-0.630739000	1.856583000
8	1.964827000	1.842296000	0.885088000
6	-0.053825000	2.256767000	-0.812683000
1	0.909130000	2.616886000	-1.174485000
1	-0.731282000	2.130569000	-1.655533000
1	-0.465858000	3.000244000	-0.127808000
6	-2.123540000	-0.186727000	0.085147000
6	-2.015521000	-0.231160000	1.617329000
1	-1.485414000	0.642654000	2.001965000
1	-3.014530000	-0.239810000	2.060861000
1	-1.497219000	-1.127774000	1.968982000
6	-2.925878000	1.058579000	-0.335732000
1	-2.504488000	1.976808000	0.071469000
1	-2.985757000	1.156299000	-1.423769000
1	-3.947596000	0.971178000	0.042460000
6	-2.879416000	-1.431057000	-0.431770000
1	-3.925535000	-1.381508000	-0.121886000
1	-2.870432000	-1.487499000	-1.526113000
1	-2.481930000	-2.367408000	-0.028786000
1	-0.804609000	-0.101459000	-1.659495000

21) Cartesian coordinates from optimized structures of I_z.

Figure S17. Cartesian coordinates from optimized structure of the $\mathbf{I_z}$ at the B3LYP/6-311++G** level of theory.

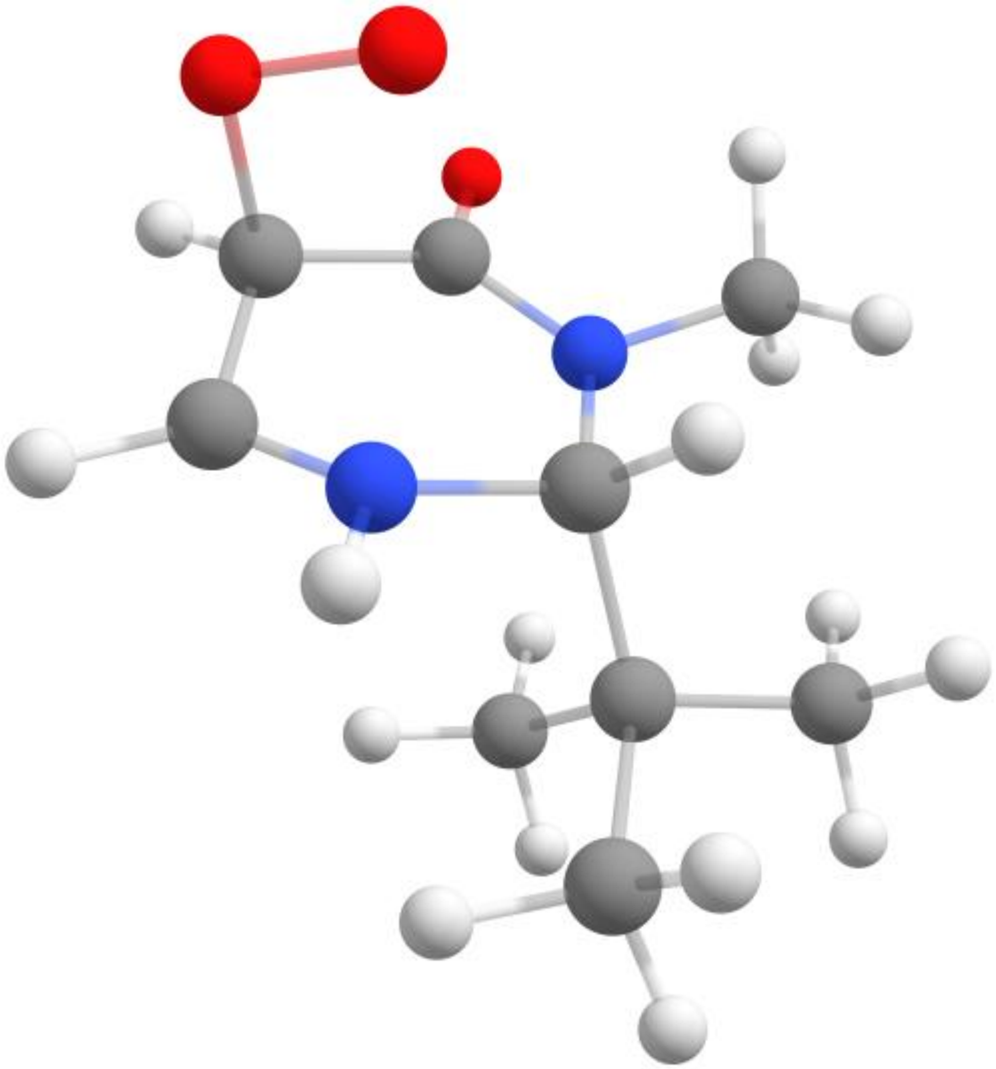

E = -687.578826

Negative eigenvalues = 0

7	0.067430000	-1.429781000	-0.202479000
6	-0.586257000	-0.157893000	-0.532443000
7	0.237332000	0.976607000	-0.118301000
6	1.426637000	0.879702000	0.560591000
6	1.950582000	-0.517572000	0.817693000
6	0.964723000	-1.592734000	0.749708000
1	-0.358902000	-2.243748000	-0.621931000

8	2.726368000	-0.994856000	-0.354029000
8	2.086056000	-0.736401000	-1.542248000
1	1.203093000	-2.595536000	1.077063000
1	2.630681000	-0.537445000	1.662918000
8	2.076168000	1.858754000	0.883211000
6	-0.000891000	2.273587000	-0.765733000
1	0.960102000	2.689338000	-1.067094000
1	-0.624293000	2.130066000	-1.646349000
1	-0.483454000	2.985395000	-0.092449000
6	-2.051281000	-0.156705000	0.073009000
6	-2.015405000	-0.078504000	1.607199000
1	-1.536516000	0.842363000	1.947126000
1	-3.034288000	-0.093231000	2.004191000
1	-1.477908000	-0.921962000	2.047985000
6	-2.863335000	1.031371000	-0.480064000
1	-2.498243000	1.990671000	-0.115926000
1	-2.861360000	1.051908000	-1.574224000
1	-3.903271000	0.936371000	-0.156485000
6	-2.770153000	-1.450451000	-0.371279000
1	-3.828590000	-1.393400000	-0.106941000
1	-2.715638000	-1.594105000	-1.456382000
1	-2.372653000	-2.342696000	0.119867000
1	-0.668552000	-0.136549000	-1.623465000

22) Cartesian coordinates from optimized structures of I_D.

Figure S18. Cartesian coordinates from optimized structure of the $\mathbf{I_D}$ at the B3LYP/6-311++G** level of theory.

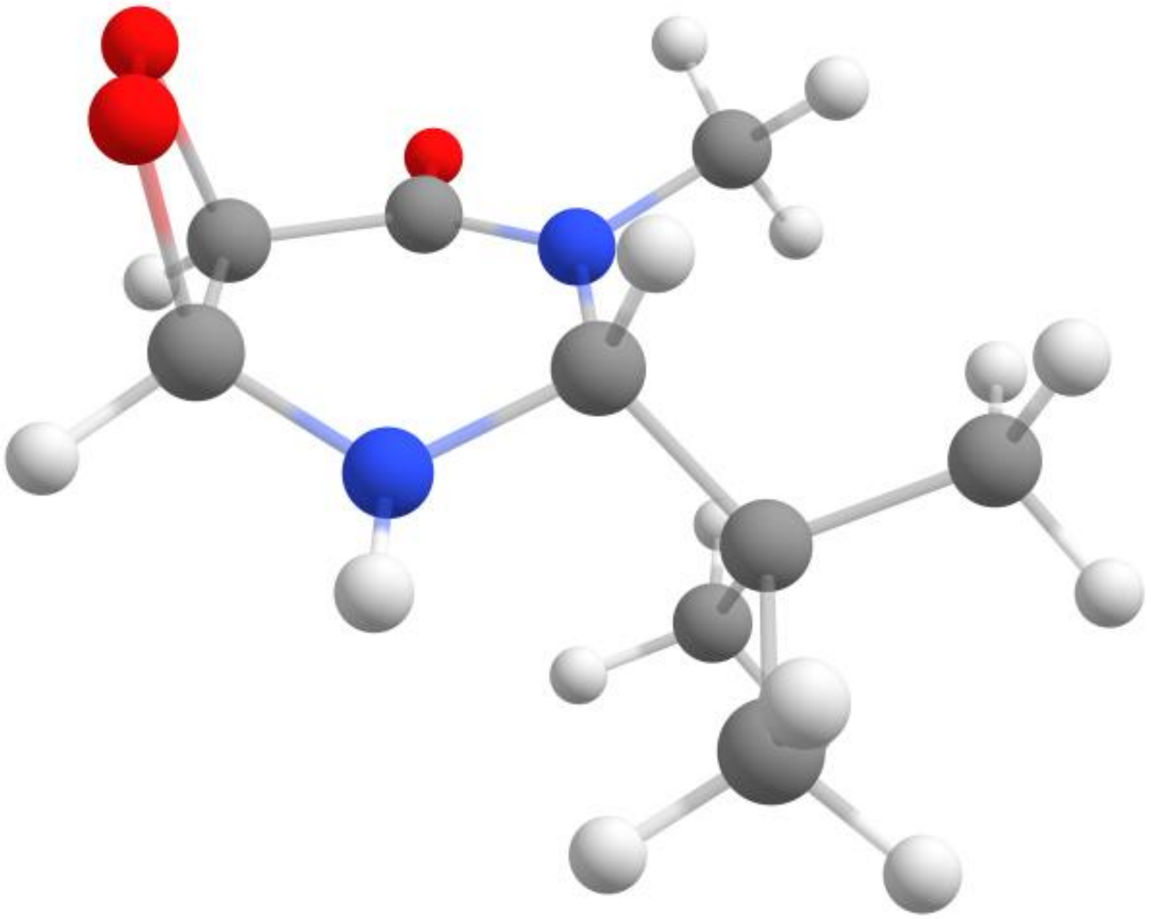

E = -687.622762

Negative eigenvalues = 0

7	0.043125465	-1.380461731	0.329671458
6	-0.598716680	-0.286547555	-0.398771752
7	0.169276000	0.957628000	-0.183078000
6	1.384991000	1.045976000	0.448863000
6	2.181696000	-0.234723000	0.642752000
6	1.436557000	-1.509519000	0.331675000
1	-0.438156000	-2.255633000	0.190874000
8	3.019715000	-0.441666000	-0.531068000
8	2.127000000	-1.545467000	-0.982802000

1	1.725714000	-2.391809000	0.906109000
1	2.775724000	-0.176925000	1.553829000
8	1.910754000	2.113481000	0.725936000
6	-0.274516000	2.182264000	-0.855065000
1	0.598042000	2.808865000	-1.026950000
1	-0.732431000	1.925781000	-1.810881000
1	-0.984925000	2.755163000	-0.254579000
6	-2.111847000	-0.215308000	0.061690000
6	-2.216392000	0.326919000	1.495653000
1	-1.860431000	1.357069000	1.565698000
1	-3.258186000	0.309869000	1.828586000
1	-1.628829000	-0.279858000	2.188008000
6	-2.968649000	0.639775000	-0.898033000
1	-2.771216000	1.706297000	-0.817052000
1	-2.823097000	0.341390000	-1.941220000
1	-4.025909000	0.492841000	-0.662197000
6	-2.711647000	-1.642367000	0.011279000
1	-3.792224000	-1.586394000	0.159947000
1	-2.544449000	-2.121787000	-0.960003000
1	-2.322668000	-2.291092000	0.799478000
1	-0.592387000	-0.483544000	-1.482585000

23) Supplementary References

1. O. V. Dolomanov, L. J. Bourhis, R. J. Gildea, J. A. K. Howard and H. Puschmann, *Journal of Applied Crystallography*, **2009**, *42*, 339–341.
2. G. M. Sheldrick, *Acta Crystallogr., Sect. A: Found. Adv.*, **2015**, *71*, 3–8.
3. G. M. Sheldrick, *Acta Cryst. C*, **2015**, *71*, 3–8.
4. Méndez Ochoa, Adrian. Síntesis y resolución de 4-quinazolinonas y su posible utilidad en la síntesis de ácidos β-aminociclohexancarboxílicos MSc Thesis Dissertation, Autonomous Morelos State University, **2017**.
